# CLPX regulates mitochondrial fatty acid β-oxidation in liver cells

**DOI:** 10.1016/j.jbc.2023.105210

**Published:** 2023-09-03

**Authors:** Ko Suzuki, Yoshiko Kubota, Kiriko Kaneko, Costantine Chasama Kamata, Kazumichi Furuyama

**Affiliations:** Department of Molecular Biochemistry, Iwate Medical University, Yahaba, Iwate, Japan

**Keywords:** beta-oxidation, mitochondria, protein‒protein interaction, hepatocyte, glucagon, CLPX

## Abstract

Mitochondrial fatty acid oxidation (β-oxidation) is an essential metabolic process for energy production in eukaryotic cells, but the regulatory mechanisms of this pathway are largely unknown. In the present study, we found that several enzymes involved in β-oxidation are associated with CLPX, the AAA+ unfoldase that is a component of the mitochondrial matrix protease ClpXP. The suppression of CLPX expression increased β-oxidation activity in the HepG2 cell line and in primary human hepatocytes without glucagon treatment. However, the protein levels of enzymes involved in β-oxidation did not significantly increase in CLPX-deleted HepG2 cells (CLPX-KO cells). Coimmunoprecipitation experiments revealed that the protein level in the immunoprecipitates of each antibody changed after the treatment of WT cells with glucagon, and a part of these changes was also observed in the comparison of WT and CLPX-KO cells without glucagon treatment. Although the exogenous expression of WT or ATP-hydrolysis mutant CLPX suppressed β-oxidation activity in CLPX-KO cells, glucagon treatment induced β-oxidation activity only in CLPX-KO cells expressing WT CLPX. These results suggest that the dissociation of CLPX from its target proteins is essential for the induction of β-oxidation in HepG2 cells. Moreover, specific phosphorylation of AMP-activated protein kinase and a decrease in the expression of acetyl-CoA carboxylase 2 were observed in CLPX-KO cells, suggesting that CLPX might participate in the regulation of the cytosolic signaling pathway for β-oxidation. The mechanism for AMP-activated protein kinase phosphorylation remains elusive; however, our results uncovered the hitherto unknown role of CLPX in mitochondrial β-oxidation in human liver cells.

Mitochondria are essential intracellular organelles in eukaryotic cells since they are the sites of several types of energy production metabolism, such as the tricarboxylic acid cycle, oxidative phosphorylation, and fatty acid oxidation (β-oxidation) (1, 2). Therefore, previous research has focused on mitochondrial dysfunction in patients with cancer, neurodegenerative diseases, or diabetes mellitus (3, 4, 5). Similar to the processes in other intracellular organelles, the function of mitochondria is maintained by the quality control of mitochondrial proteins mediated by specific molecular chaperones and protein degradation pathways (6). ClpXP and LONP1 are mitochondrial matrix AAA+ proteases that contribute to the maintenance of protein homeostasis by degrading their substrate proteins (7, 8). It has been suggested that LONP1 mainly degrades oxidized proteins (9, 10), whereas ClpXP is involved in the degradation of unfolded proteins in eukaryotic cells. These proteases may cooperate with each other to control protein homeostasis in mitochondria (11). In fact, Lee *et al*. reported that ClpXP and LONP1 cooperatively regulate the survival of cancer cells (12). We also reported that excess heme stimulates the rapid degradation of nonspecific 5-aminolevulinate synthase (ALAS1) protein, the rate-limiting enzyme for heme synthesis, by enhancing the association of ClpXP and ALAS1 (13), although oxidized ALAS1 is preferentially degraded by LONP1 (14).

The protease ClpXP consists of a hexamer of ClpX and a heptamer of ClpP (15), and it has been suggested that ClpX recognizes the substrate protein and uses ATP to transfer it to ClpP for digestion (16). Substrates or binding proteins of ClpXP have been reported in both prokaryotes (17, 18) and eukaryotes (19, 20, 21). Using mammalian mitochondria isolated from mouse embryonic fibroblasts of ClpP KO mice, Szczepanowska *et al*. (22) identified 66 proteins as potential candidate substrates for ClpXP by substrate-trapping screen. Of these 66 proteins, 15 proteins are orthologs of known bacterial ClpXP substrates, and the other proteins are involved in metabolic processes in mitochondria, such as the translation pathway (eleven proteins), respiratory chain (eleven proteins), amino acid metabolism (eight proteins), fatty acid metabolism (six proteins), and TCA cycle (three proteins). These proteins are candidates for the substrate of ClpXP protease. However, researchers have recently focused on the specific role of ClpX in mammalian cells. Reportedly, ClpX activates the Alas protein by inserting a cofactor into the protein in yeast mitochondria in which the ortholog of ClpP was not expressed. Thus, the authors concluded that this function is independent of the proteolytic activity of ClpP (21). Furthermore, Al-Furoukh *et al*. (19) reported that overexpression of ClpX in myoblasts could trigger the mitochondrial unfolded protein response in mammalian myoblasts. These reports suggested that ClpX plays important roles as a chaperone protein in eukaryotic cells.

In the present study, we sought to identify proteins interacting with CLPX in human fibroblasts using LC–MS/MS proteomic and bioinformatic analyses, and we successfully identified several proteins that interact with the CLPX protein. Among these interactors, we focused on proteins involved in fatty acid oxidation (β-oxidation) in mitochondria because the knockout of CLPX in these cells did not affect the expression level of these interactors, suggesting that the transfer of these proteins to CLPP for proteolysis is not the major function of CLPX in human mitochondria. The complex formation between CLPX and these proteins was confirmed in HepG2 hepatoma cells using immunoprecipitation. In HepG2 cells, knockout or knockdown of CLPX increases β-oxidation activity, and exogenous expression of CLPX is able to attenuate this phenomenon in CLPX-KO cells. We further analyzed the role of CLPX in β-oxidation, and our results revealed an unknown function of CLPX in mitochondrial energy metabolism.

## Results

### CLPX associates with several enzymes catalyzing β-oxidation

**Figure 1:**
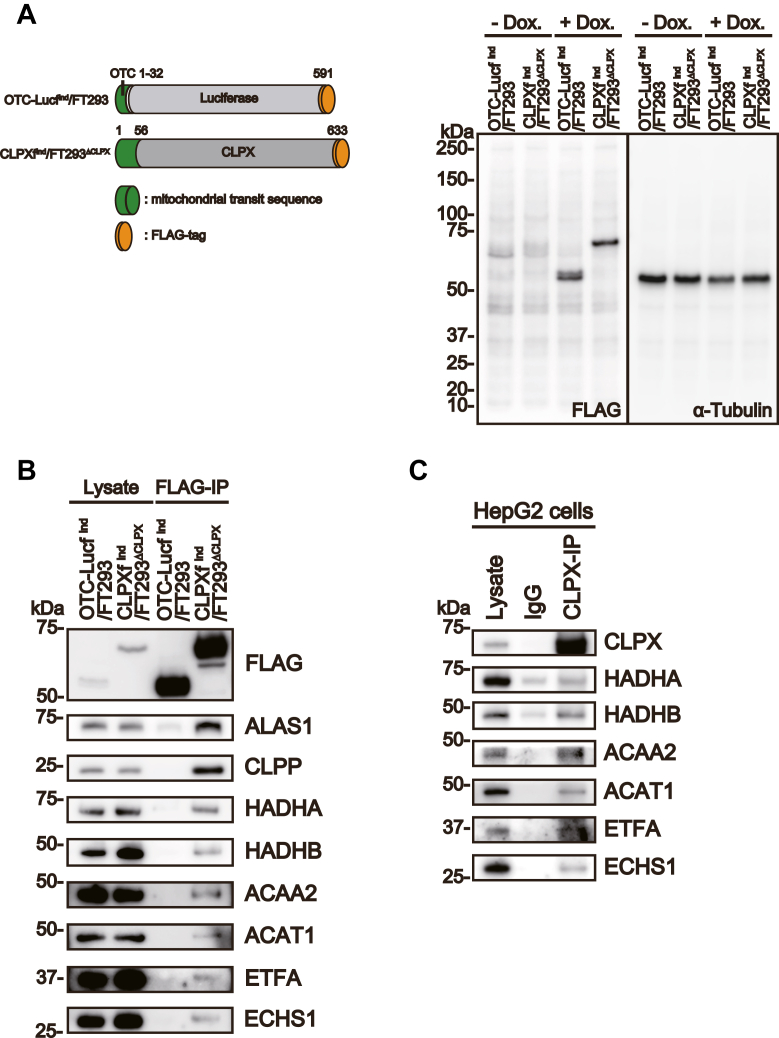
**Proteomic and bioinformatic analyses for identification of binding proteins for mitochondrial matrix proteases.***A*, *left panel*: Structure of the FLAG-tagged protein. OTC-Lucf^ind^/FT293 consisted of the N-terminal region of ornithine transcarbamylase, full-length luciferase, and a FLAG sequence at the C terminus. CLPXf^ind^/FT293^△CLPX^ consisted of full-length CLPX and a FLAG sequence at the C terminus. *Right panel*: Western blot data showing the doxycycline-inducible expression of the FLAG-tagged protein in each clone. *B*, validation of the protein binding between FLAG-tagged CLPX and the proteins involved in fatty acid oxidation in FT293 cells. Five micrograms of each protein sample was subjected to SDS–PAGE and then analyzed by Western blotting. ALAS1 and CLPP were used as positive control CLPX-binding proteins. OTC-Lucf was used as a negative control. *C*, validation of protein binding of endogenous CLPX and proteins involved in β-oxidation. Rabbit IgG was used as a negative control for immunoprecipitation.

To identify proteins that bind CLPX, we established the genetically modified FT293 cell line FT293^ΔCLPX^, in which endogenous CLPX was deleted using genome editing (13). Then, an expression cassette of FLAG-tagged CLPX (CLPXf) protein was introduced into FT293^ΔCLPX^ to establish CLPXf^ind^/FT293^ΔCLPX^, in which the FLAG-tagged CLPX protein at its C-terminal end was expressed in a doxycycline-inducible manner (Fig. 1*A*). To determine the nonspecifically interacting proteins, we used a chimeric protein composed of the mitochondrial transit peptide region of ornithine transcarbamylase (OTC) and Flag-tagged luciferase (OTC-Lucf^ind^/FT293, Fig. 1*A*). The FLAG-tagged proteins were immunoprecipitated using anti-DDDDK-tag antibody-conjugated agarose beads, and the immunoprecipitated proteins were eluted from the agarose beads by DDDDK peptides. The eluted precipitates were digested with trypsin and analyzed by LC–MS/MS and with the MASCOT database (23). To extract mitochondrial proteins, each dataset was further analyzed with DAVID Bioinformatics Resources v.6.8 (24, 25). As shown in Table 1, the CLPXf precipitants included 27 specific mitochondrial-binding proteins. These proteins include aconitate hydratase (ACO2), citrate synthase, and isocitrate dehydrogenase [NAD] subunit alpha (IDH3), which have been reported to be copurified with ClpXP in eukaryotes (20). The recently identified CLPX-binding protein polymerase delta-interacting protein 2 (POLDIP2) (26) and coiled-coil-helix-coiled-coil-helix domain-containing protein 2 (CHCHD2) (22) were also included. Identification of these proteins confirmed that our immunoprecipitation experiments were appropriately performed. In addition to these previously reported ClpXP-interacting proteins, pyrroline-5-carboxylate reductase 2 (PYCR2), the genetic mutation of which causes microcephaly (27), was also identified. The mass spectrometry data were deposited at the Proteome Xchange Consortium (ID: PXD032346).

**Table 1:** Identified mitochondrial proteins in CLPXf^ind^/FT293^ΔCLPX^ cells

Specific binding proteins for CLPX				
UniProt ID	Gene name	Protein name	Unique peptides	Peptide sequence matches
P42765	*ACAA2*	3-Ketoacyl-CoA thiolase, mitochondrial	7	10
P24752	*ACAT1*	Acetyl-CoA acetyltransferase, mitochondrial	2	2
Q99798	*ACO2*	Aconitate hydratase, mitochondrial	2	6
O00154	*ACOT7*	Cytosolic acyl coenzyme A thioester hydrolase	1	6
P54819	*AK2*	Adenylate kinase 2, mitochondrial	2	3
Q8N5M1	*ATPAF2*	ATP synthase mitochondrial F1 complex assembly factor 2	3	7
Q9Y6H1	*CHCHD2*	Coiled-coil-helix-coiled-coil-helix domain-containing protein 2	5	7
Q5T1J5	*CHCHD2P9*	Putative coiled-coil-helix-coiled-coil-helix domain-containing protein CHCHD2P9, mitochondrial	4	5
P12532	*CKMT1A*	Creatine kinase U-type, mitochondrial	5	5
O75390	*CS*	Citrate synthase, mitochondrial	3	14
P63167	*DYNLL1*	Dynein light chain 1, cytoplasmic	1	3
P30084	*ECHS1*	Enoyl-CoA hydratase, mitochondrial	7	29
P13804	*ETFA*	Electron transfer flavoprotein subunit alpha, mitochondrial	5	8
P40939	*HADHA*	Trifunctional enzyme subunit alpha, mitochondrial	10	17
P55084	*HADHB*	Trifunctional enzyme subunit beta, mitochondrial	2	2
P50213	*IDH3A*	Isocitrate dehydrogenase [NAD] subunit alpha, mitochondrial	1	6
Q92552	*MRPS27*	28S ribosomal protein S27, mitochondrial	2	2
P52815	*MRPL12*	39S ribosomal protein L12, mitochondrial	2	7
P00403	*MT-CO2*	Cytochrome c oxidase subunit 2	3	4
Q96DU9	*PABPC5*	Polyadenylate-binding protein 5	1	5
P11177	*PDHB*	Pyruvate dehydrogenase E1 component subunit beta, mitochondrial	2	2
P36873	*PPP1CC*	Serine/threonine-protein phosphatase PP1-gamma catalytic subunit	4	6
Q96C36	*PYCR2*	Pyrroline-5-carboxylate reductase 2	25	30
Q9H9B4	*SFXN1*	Sideroflexin-1	1	4
P00441	*SOD1*	Superoxide dismutase [Cu-Zn]	3	4
Q9UJZ1	*STOML2*	Stomatin-like protein 2, mitochondrial	3	7
Q16740	*CLPP*	ATP-dependent Clp protease proteolytic subunit, mitochondrial	52	56
O76031	*CLPX*	ATP-dependent Clp protease ATP-binding subunit clpX-like, mitochondrial	531	729

To annotate the intracellular functions of the mitochondrial proteins identified in our proteomic analysis, we further performed bioinformatic analysis using DAVID Bioinformatics Resources v.6.8. As shown in Table 2, this analysis revealed that the proteins were associated with the “Biosynthesis of antibiotics”, “Carbon metabolism,” and “Metabolic pathway” terms. Carbon metabolism and metabolic pathways are key pathways of mitochondrial function, and it has been suggested that fungal ClpXP might be involved in fatty acid metabolism based on the results of a proteomic analysis (20). Furthermore, analyses of the phenotype of ClpP KO mice revealed the unknown function of ClpP in the energy metabolism of mammals since ClpP KO mice are protected from high-fat diet–induced obesity, insulin resistance, and glucose intolerance (28, 29). Thus, we decided to focus on proteins associated with the “Metabolic pathway” term that were involved in fatty acid metabolism or β-oxidation. Western blot analysis of the immunoprecipitates of the FLAG-tagged protein validated the interaction of CLPXf with β-oxidation–related proteins in FT293 cells, consistent with the results of LC–MS/MS analysis (Fig. 1*B*). To obtain more detailed insights into the relationship between CLPX and β-oxidation, we used hepatoblastoma-derived HepG2 cells instead of kidney-derived FT293 cells for further analyses since the β-oxidation pathway could be activated in liver cells by glucagon stimulation. Immunoprecipitation using an anti-CLPX antibody followed by Western blot analyses confirmed the interaction of endogenous CLPX with several proteins catalyzing β-oxidation in HepG2 cells (Fig. 1*C*). This result confirms that these proteins bind to endogenous CLPX in HepG2 cells.

**Table 2:** Predicted intracellular function of CLPX from identified mitochondrial proteins and DAVID analysis

GO annotation in CLPX-binding proteins			
Category	Term		
Cluster 1	Enrichment score: 7.48	Count	FDR
KEGG_PATHWAY	hsa01130:Biosynthesis of antibiotics	11	2.21E-08
KEGG_PATHWAY	hsa01200:Carbon metabolism	7	2.71E-04
KEGG_PATHWAY	hsa01100:Metabolic pathways	13	0.005894


Category	Term		
Cluster 2	Enrichment score: 4.05	Count	FDR
KEGG_PATHWAY	hsa01130:Biosynthesis of antibiotics	11	2.21E-08
KEGG_PATHWAY	hsa00062:Fatty acid elongation	5	4.01E-04
GOTERM_BP_DIRECT	GO:0006635∼fatty acid beta-oxidation	5	7.22E-04
KEGG_PATHWAY	hsa00071:Fatty acid degradation	5	0.003448
KEGG_PATHWAY	hsa00280:Valine, leucine, and isoleucine degradation	5	0.005450
KEGG_PATHWAY	hsa01212:Fatty acid metabolism	5	0.005936
GOTERM_MF_DIRECT	GO:0003988∼acetyl-CoA C-acyltransferase activity	3	0.015797
INTERPRO	IPR020610:Thiolase, active site	3	0.020476
INTERPRO	IPR020615:Thiolase, acyl-enzyme intermediate active site	3	0.020476
INTERPRO	IPR002155:Thiolase	3	0.030686
INTERPRO	IPR020613:Thiolase, conserved site	3	0.030686
INTERPRO	IPR020616:Thiolase, N-terminal	3	0.030686
INTERPRO	IPR020617:Thiolase, C-terminal	3	0.030686
GOTERM_MF_DIRECT	GO:0004300∼enoyl-CoA hydratase activity	3	0.039409
UP_SEQ_FEATURE	active site:Acyl-thioester intermediate	3	0.046962


Category	Term		
Cluster 3	Enrichment score: 3.93	Count	FDR
KEGG_PATHWAY	hsa01200:Carbon metabolism	7	2.71E-04
UP_KEYWORDS	Tricarboxylic acid cycle	4	0.004432
GOTERM_BP_DIRECT	GO:0006099∼tricarboxylic acid cycle	4	0.014399

### Knockout of *CLPX* induces β-oxidation in HepG2 cells

**Figure 2:**
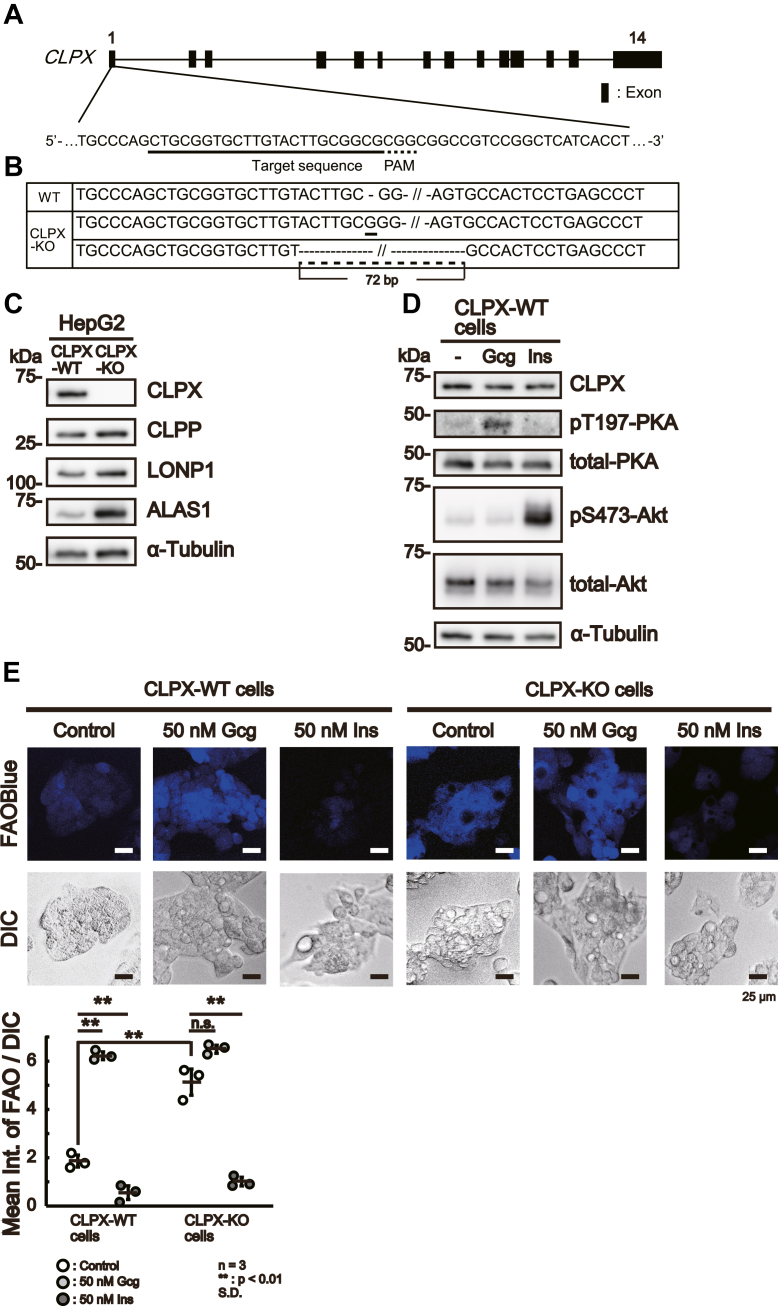

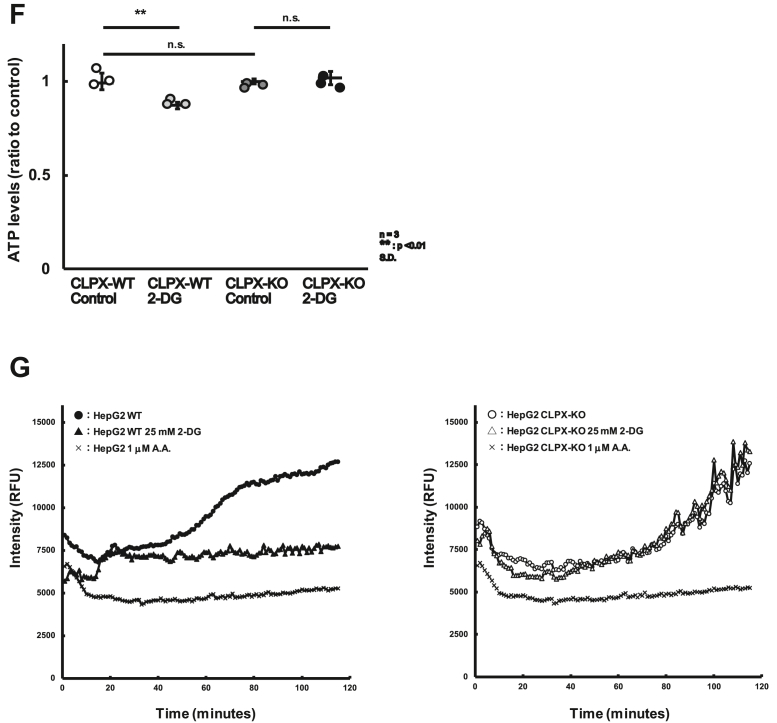
**Upregulation of β-oxidation in CLPX-KO HepG2 cells.***A*, schematic presentation of the human *CLPX* gene. A portion of the first exon of the *CLPX* gene is enlarged to indicate the Cas9 target sequence and the protospacer adjacent motif (PAM). The underlined and dotted underlined sequences indicate the Cas9 target sequence and PAM, respectively. *B*, DNA sequences of mutant clones. The insertion of guanine (NC_000015.10:g.65185126_65185127insC, NM_006660.5:c.216_217 insG) or deletion of 71 base pairs (NC_000015.10:g.65185062_65185132del) is indicated by bold underline or dotted underline, respectively. The hyphens in the sequences of the mutant clones indicate the deleted sequences. *C*, validation of CLPX knockout in HepG2 cells by Western blot analysis. Proteins from cell lysates of CLPX-WT cells or CLPX-KO cells were examined by Western blotting. Five micrograms of each protein was subjected to SDS–PAGE followed by Western blotting. *D*, the activation of intracellular signaling by Gcg or Ins was validated by detecting the phosphorylation of target proteins. CLPX-WT (HepG2) cells were pretreated with fatal bovine serum (FBS)-free culture medium for 12 h and then treated with 50 nM Gcg or 50 nM Ins for 3 h. After treatment, each cell line was lysed as described in the Experimental procedures. Five micrograms of protein was subjected to SDS–PAGE and Western blotting. Gcg treatment and Ins treatment clearly induced the phosphorylation of T197-PKA and S473-Akt, respectively. *E*, detection of β-oxidation activity using the fluorescent probe FAOBlue. CLPX-WT and CLPX-KO cells were treated with 50 nM Gcg or 50 nM Ins for 3 h and then treated with 10 μM FAOBlue for 30 min. The β-oxidation activity was visualized by fluorescence of the probe (FAOBlue). The DIC image shows the shape of each cell. β-oxidation activity was quantified as described in the Experimental procedures. The mean FAO-int per DIC-area (Mean Int. of FAO/DIC) is indicated on the Y axis. The error bars indicate the SDs from three independent experiments. ∗∗*p* < 0.01 (one-way ANOVA with Tukey’s post hoc test). Each result was calculated using data from more than three independent experiments. *F*, under basal conditions, there was no significant difference in intracellular ATP levels between CLPX-WT (CLPX-WT Control) and CLPX-KO cells (CLPX-KO Control). After treatment with 25 mM 2-deoxy-D-glucose (2-DG), intracellular ATP levels were significantly decreased in CLPX-WT cells (CLPX-WT 2-DG), whereas they were not decreased in CLPX-KO cells (CLPX-WT 2-DG). *G*, in the basal state, the difference in the cumulative fluorescence intensity between CLPX-WT cells (*left panel*, *closed circle*) and CLPX-KO cells (*right panel*, *open circle*) was not clear 120 min after the initiation of the experiment. CLPX-WT cells showed decreased oxygen consumption (*left panel*, *closed triangle*) after treatment with 25 mM 2-DG compared with that in the basal state; however, no clear difference in oxygen consumption (*right panel*, *open triangle* for 2-DG treatment) was found between 2-DG–treated and untreated CLPX-KO cells.

To determine the role of CLPX in β-oxidation in HepG2 cells, we knocked out the *CLPX* gene in HepG2 cells using genome editing (Fig. 2*A*) and successfully established CLPX-null cells (designated CLPX-KO cells). The genotype of the CLPX-KO cells was confirmed (Fig. 2*B*). As shown in Figure 2*C*, the CLPX protein was undetectable in CLPX-KO cells using Western blot analysis while it was clearly detectable in WT HepG2 (hereafter CLPX-WT) cells. As we have reported previously using FT293 cells (13), ALAS1 protein levels were higher in CLPX-KO cells than in CLPX-WT cells, even though the levels of CLPP, the catalytic subunit of ClpXP, were slightly increased in CLPX-KO cells (Fig. 2*C*). Moreover, the levels of another AAA+ mitochondrial matrix protease, LONP1, were also slightly increased in CLPX-KO cells. These results suggest that CLPX, but not LONP1, regulates the steady-state levels of ALAS1 in HepG2 cells.

Then, we examined the β-oxidation activity in these cells using a recently developed sensitive probe for the detection of mitochondrial β-oxidation, FAOBlue (30). First, we treated CLPX-WT cells with 50 nM glucagon (Gcg) (31) or 50 nM insulin (Ins) (32) to induce or repress β-oxidation, respectively, and then assessed the intracellular signaling mechanisms of these hormones. As shown in Figure 2*D*, we confirmed the occurrence of increased phosphorylation of T197-PKA in Gcg-treated cells and increased phosphorylation of S473-Akt in Ins-treated cells, which indicated activation of Gcg and Ins signaling, respectively. Then, we determined β-oxidation activity in these cells using FAOBlue. Gcg treatment induced β-oxidation in CLPX-WT cells, although Ins treatment reduced β-oxidation activity in CLPX-WT cells (Fig. 2*E*). These results suggest that FAOBlue specifically detects β-oxidation in mitochondria rather than in peroxisomes and that Gcg can induce β-oxidation activity in HepG2 cells in the presence of endogenous CLPX. Interestingly, we found that CLPX-KO cells exhibited greater β-oxidation activity than CLPX-WT cells under basal conditions (Fig. 2*E*, controls). Quantitative analysis of the intensity of FAOBlue fluorescence revealed that CLPX-KO cells showed 2.7 times higher β-oxidation activity than CLPX-WT cells without any treatment (Fig. 2*E*, graph). Although Gcg treatment further increased the β-oxidation activity in CLPX-KO cells to a level similar to that observed in Gcg-stimulated CLPX-WT cells, the difference was not statistically significant (Fig. 2*E*, CLPX-KO cells). On the other hand, Ins treatment reduced β-oxidation activity even in CLPX-KO cells, consistent with previous findings that β-oxidation is suppressed by Ins treatment (33). These results suggested that the β-oxidation activity was already increased to almost the maximum level in CLPX-KO cells without any stimulation, whereas the Gcg and Ins signaling pathways remained intact in these cells. Moreover, it is also suggested that the downstream of the Gcg signaling pathway might be activated in CLPX-KO cells, since Gcg treatment did not have an obvious additive effect on β-oxidation activity and Ins stimulation reduced β-oxidation activity in CLPX-KO cells.

To confirm the activation of β-oxidation in CLPX-KO cells, we measured ATP production and oxygen consumption in both CLPX-KO and CLPX-WT cells with and without 2-deoxy-D-glucose (2-DG), a specific inhibitor of glycolysis, in the culture medium. As shown in Figure 2*F*, 2-DG treatment reduced ATP production in CLPX-WT cells but did not affect ATP production in CLPX-KO cells. Similarly, 2-DG treatment inhibited the increase in oxygen consumption in CLPX-WT cells (Fig. 2*G*, left panel) but did not affect oxygen consumption in CLPX-KO cells (Fig. 2*G*, right panel). Oxygen consumption did not increase in either cell line under antimycin A treatment. These results suggest that glycolysis is activated in CLPX-WT cells to obtain ATP and supply acetyl-CoA for oxidative phosphorylation under steady-state conditions, whereas CLPX-KO cells depend on oxidative phosphorylation to produce ATP. Taken together with the results obtained using FAO fluorescent dye, our data strongly suggest that β-oxidation activity is induced in CLPX-KO cells in the absence of glucagon stimulation.

### CLPX regulates β-oxidation in hepatocarcinoma cells as well as in normal human hepatocytes

**Figure 3:**
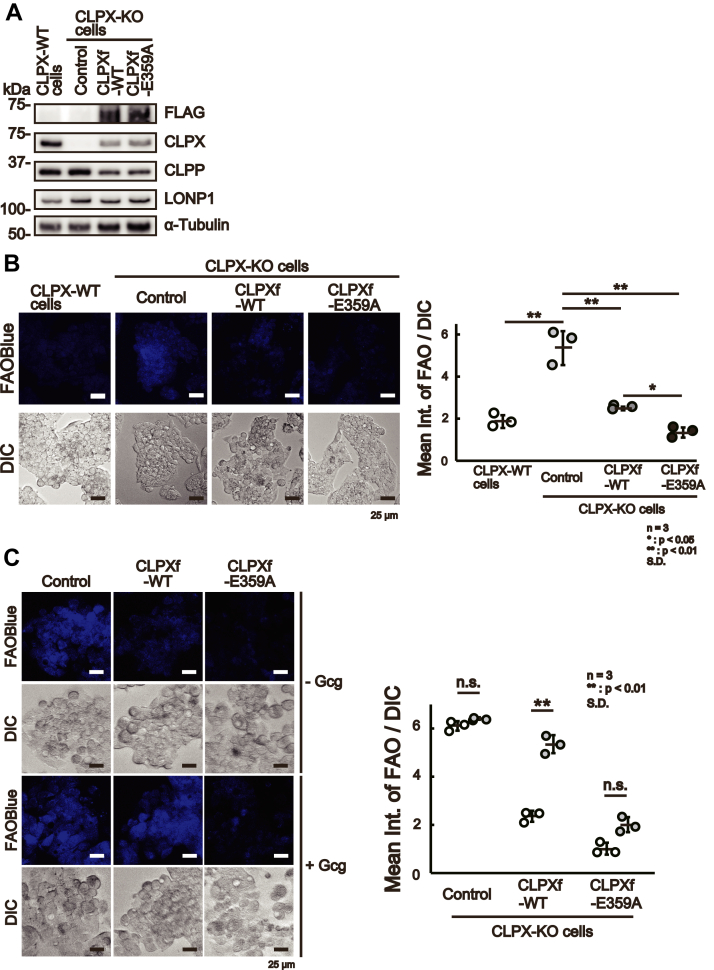

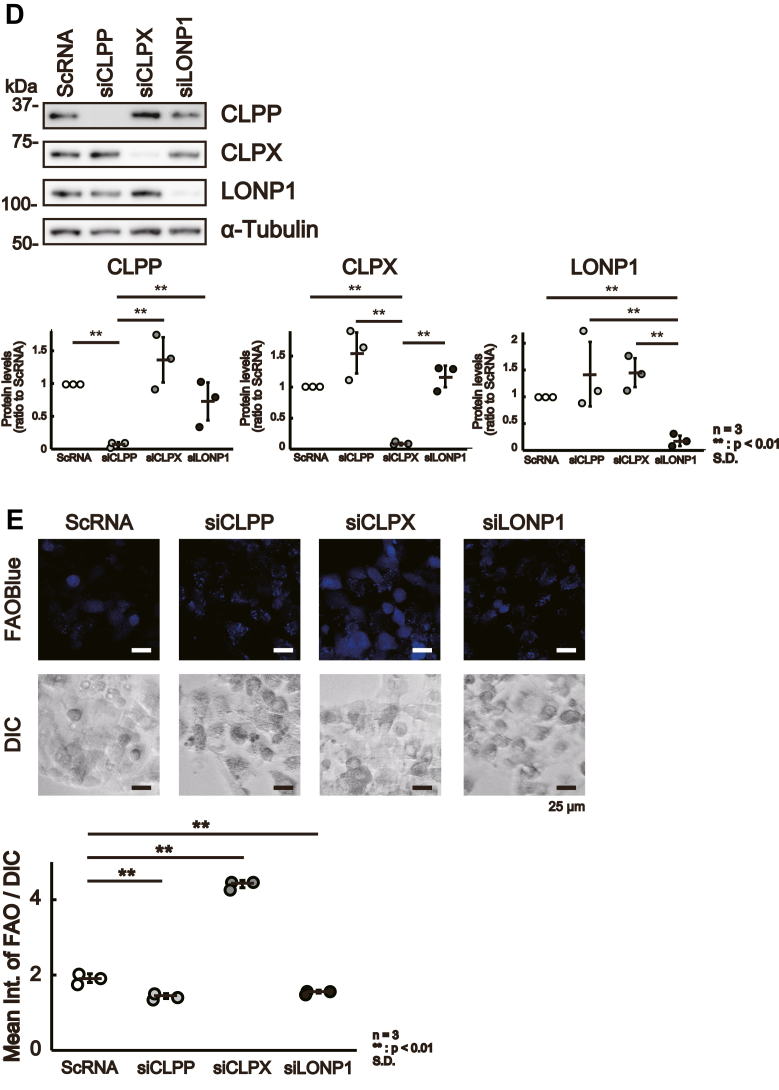

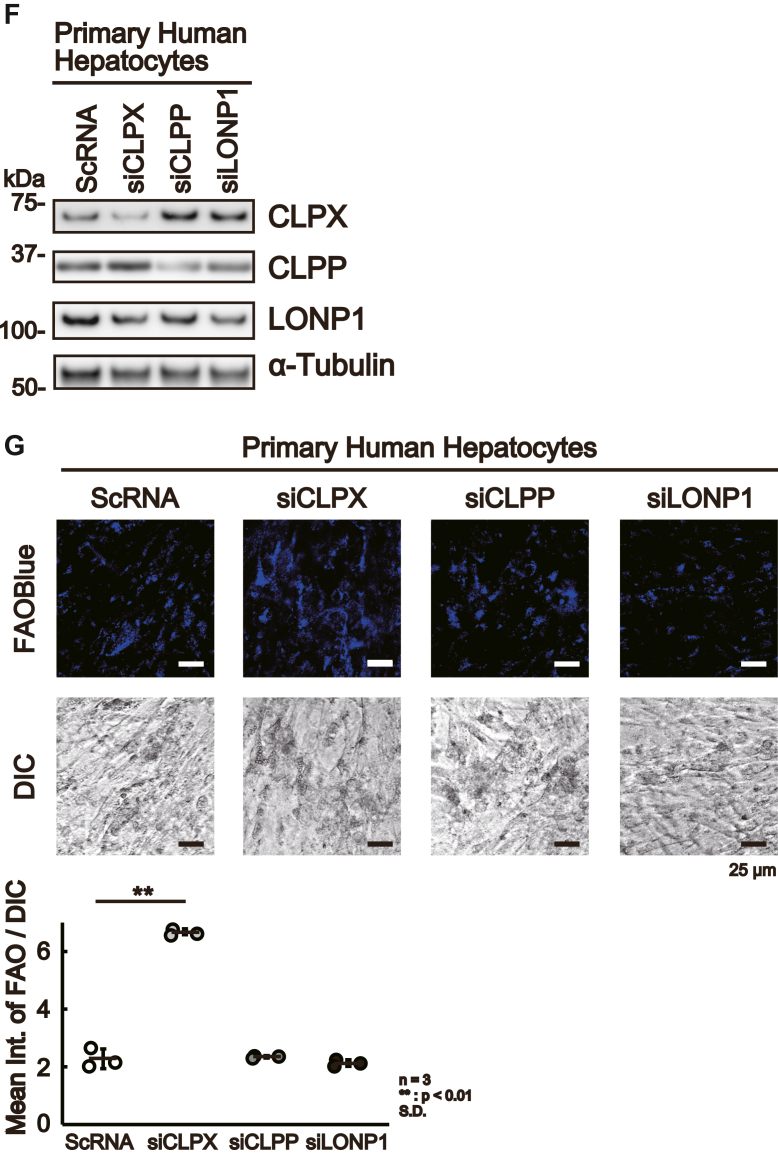
**CLPX regulates β-oxidation in liver cells.***A*, expression of CLPX restored the regulation of β-oxidation in CLPX-KO cells. Empty vector (control), CLPXf-WT expression vector, or CLPXf-E359A expression vector was transfected into CLPX-KO cells by lipofection. The expression of each protein was determined using Western blotting. *B*, the β-oxidation activity was quantified as described in the Experimental procedures. The error bars indicate the SDs from three independent experiments. ∗∗*p* < 0.01, n.s. indicates “not significant” (one-way ANOVA with Tukey’s post hoc test). *C*, mutant CLPX-expressing cells were not sensitive to Gcg. The CLPXf-WT or CLPXf-E359A expression vector was transfected into CLPX-KO cells by lipofection. Seventy-two hours after transfection, cells were treated with or without 50 nM Gcg for 3 h and then treated with 10 μM FAOBlue for 30 min, and β-oxidation activity was quantified as described in the Experimental procedures. The error bars indicate the SDs from three independent experiments. ∗∗*p* < 0.01, n.s. indicates “not significant” (one-way ANOVA with Tukey’s post hoc test). *D*, CLPX knockdown increased β-oxidation. siRNA for CLPP (siCLPP), CLPX (siCLPX), or LONP1 (siLONP1) was introduced using lipofection into CLPX-WT cells. *E*, Ninety-six hours after the introduction of each siRNA, β-oxidation was visualized with FAOBlue, and the knockdown of each protein was validated by Western blotting. *F*, siRNA for CLPP (siCLPP), CLPX (siCLPX), or LONP1 (siLONP1) was introduced using lipofection into primary human hepatocytes twice. The second transfection was performed 96 h after the initial transfection, and transfected cells were further incubated for 96 h. The effect of each siRNA was determined by Western blotting. *G*, a total of 192 h after the initial introduction of each siRNA, β-oxidation was visualized with FAOBlue, and the knockdown of each protein was validated by Western blotting. ∗∗*p* < 0.01, n.s. indicates “not significant” (one-way ANOVA with Tukey’s post hoc test).

As presented above, CLPX knockout increased β-oxidation, and the results suggest that CLPX acts as a suppressor of the spontaneous activation of β-oxidation in liver cells in the absence of exogenous inducers of β-oxidation, such as glucagon. To confirm the role of CLPX in β-oxidation, we introduced a plasmid vector expressing FLAG-tagged WT CLPX (CLPXf-WT) or ATP-hydrolysis mutant CLPX (CLPXf-E359A) into CLPX-KO cells. The mutant CLPX, CLPXf-E359A, lacked ATPase activity and maintained the CLPX interaction with its binding proteins (16); therefore, CLPXf-E359A was not able to induce CLPXP protease activity. As shown in Figure 3*A*, Western blot analysis with anti-FLAG and anti-CLPX antibodies confirmed the expression of CLPXf-WT as well as CLPXf-E359A in CLPX-KO cells. Under this condition, the expression levels of CLPP were slightly lower in CLPX-KO cells expressing CLPXf-WT or CLPXf-E359A than in nontransfected (control) CLPX-KO cells, whereas the expression levels of the mitochondrial protease LONP1 in CLPX-KO cells were not altered by the expression of CLPXf-WT or CLPXf-E359A (Fig. 3*A*). As expected, transient expression of CLPXf-WT reduced β-oxidation activity to less than half that in CLPX-KO cells (Fig. 3*B*). Interestingly, expression of the CLPXf-E359A mutant reduced β-oxidation activity more efficiently than expression of CLPXf-WT in CLPX-KO cells. The CLPX-E359A mutant binds tightly to its substrates and does not easily release them for degradation by CLPP protease (16). Thus, these results suggest that the binding function of CLPX is important for suppressing β-oxidation activity in HepG2 cells. Furthermore, we tried to stimulate β-oxidation using Gcg in WT or E359A mutant CLPXf-expressing CLPX-KO cells. Although both WT and mutant CLPXf expression reproducibly suppressed β-oxidation in CLPX-KO cells, Gcg was able to induce β-oxidation significantly only in CLPX-KO cells that expressed WT CLPXf (Fig. 3*C*). These results suggest that the binding of CLPXf to its interactors is important for the suppression of β-oxidation in untreated HepG2 cells, whereas release of interactors from CLPX is necessary for the activation of β-oxidation in HepG2 cells.

To validate the important role of CLPX in the regulation of β-oxidation in liver cells, we examined β-oxidation activity in CLPX-knockdown cells using specific siRNA. CLPP, CLPX, and LONP1 expression was independently knocked down in CLPX-WT cells by introducing siRNA specific for each mRNA (Fig. 3*D*), and β-oxidation activity was determined with FAOBlue (Fig. 3*E*). As shown in Figure 3*E*, CLPX knockdown using siRNA significantly induced β-oxidation activity in CLPX-WT cells. Again, these results confirm that CLPX protein expression is critical for the regulation of β-oxidation activity in HepG2 cells. Although the knockdown of CLPP or LONP1 marginally decreased β-oxidation activity (Fig. 3*E*), the mechanism is unclear. Since the CLPX protein level was slightly but not significantly increased in both CLPP- and LONP1-knockdown cells (Fig. 3*D*), this may affect FAO activity.

We have confirmed that Ins or Gcg appropriately regulates β-oxidation activity in HepG2 cells (Fig. 2*E*); therefore, we believe that HepG2 cells are suitable for analysis of the regulatory mechanism of β-oxidation in human liver cells. Additionally, we wanted to determine whether CLPX is involved in the regulation of β-oxidation in normal human hepatocytes. Therefore, we tried to knock down the expression of CLPX protein in primary human hepatocytes using siRNA. As shown in Figure 3*F*, siRNA against CLPX, CLPP, or LONP1 suppressed the expression of each protein, and the reduction in CLPX resulted in a significant increase in β-oxidation activity in primary human hepatocytes without Gcg stimulation. These results suggest that CLPX regulates β-oxidation activity not only in HepG2 hepatoma cells but also in human primary hepatocytes. Although β-oxidation activity was suppressed in siCLPP- or siLONP1-treated HepG2 cells (Fig. 3*E*), similar effects were not observed in primary human hepatocytes (Fig. 3*G*). In these cells, there remained a certain amount of CLPP or LONP1 protein in siCLPP- or siLONP1-treated cells, respectively (Fig. 3*F*). Thus, we need to employ a more effective method to suppress the expression of each gene in human primary hepatocytes to confirm the role of CLPP or LONP1 in the regulation of β-oxidation in primary human hepatocytes.

### Gcg regulates CLPX protein levels

**Figure 4:**
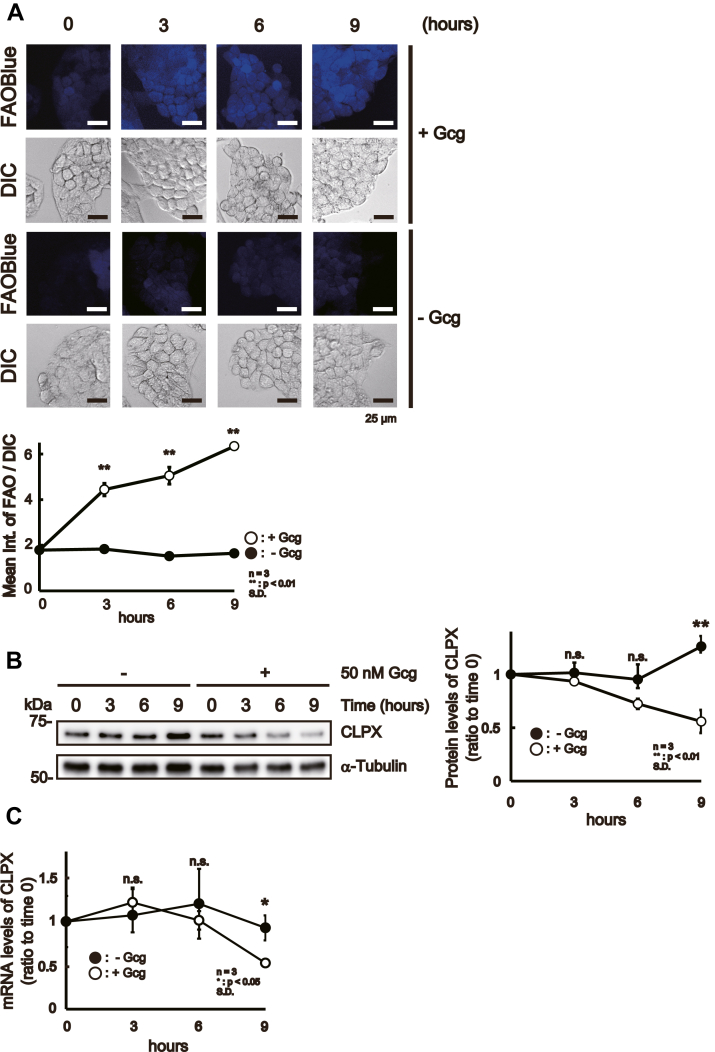

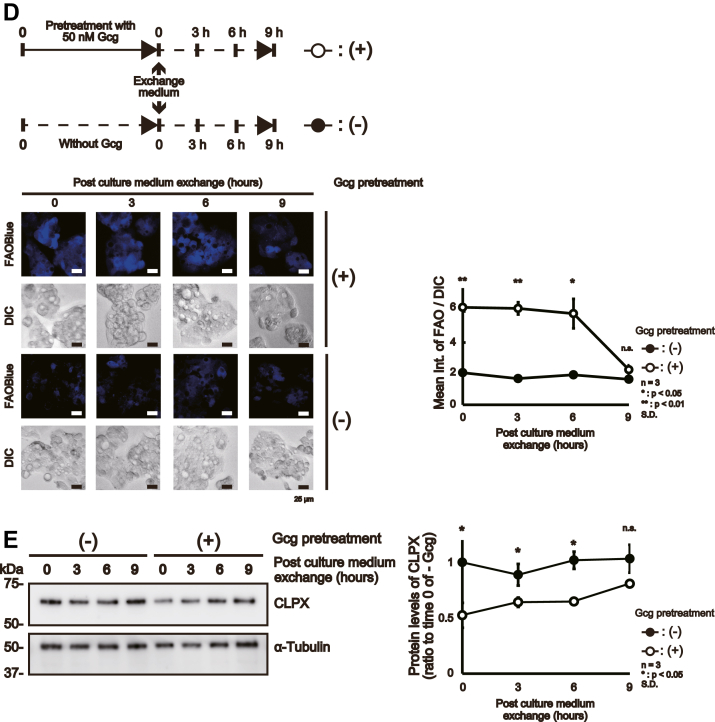
**Gcg regulates CLPX expression in HepG2 cells.** CLPX-WT cells were treated with Gcg and harvested at several time points to measure the β-oxidation activity and protein and mRNA expression levels of CLPX. CLPX-WT cells were treated with 50 nM Gcg for 3, 6, or 9 h. *A*, β-Oxidation activity was visualized at each time point with FAOBlue. *B*, CLPX protein and mRNA expression during treatment with Gcg. CLPX-WT cells were treated with 50 nM Gcg for 3, 6, or 9 h, and then total lysate or total RNA was prepared as described in the Experimental procedures. Five micrograms of protein sample was subjected to SDS–PAGE and Western blot analysis. *C*, quantitative real-time PCR analysis was performed as described in the Experimental procedures. The error bars indicate the SDs from three independent experiments. ∗*p* < 0.05 and ∗∗*p* < 0.01 (Student’s *t* test). *D*, removal of Gcg from the culture medium restored the regulation of β-oxidation by CLPX. The *upper panel* presents a scheme of the schedule of this experiment. CLPX-WT cells were treated with 50 nM Gcg for 9 h, and then the culture medium was exchanged with medium containing neither fatal bovine serum (FBS) nor Gcg. The experimental controls were cultured in medium containing neither FBS nor Gcg for 9 h. At the indicated time points, β-oxidation was visualized with FAOBlue, and samples were prepared for Western blot analysis. The lower panel shows the reduction in β-oxidation that resulted from removal of Gcg from the culture medium in a time-dependent manner. *E*, CLPX protein levels were restored after the removal of Gcg. Each result was obtained from three independent experiments.

Then, we examined whether Gcg treatment altered the expression level of CLPX in HepG2 cells during the proliferative phase. For this purpose, we treated CLPX-WT cells with 50 nM Gcg and examined the protein and mRNA expression levels of CLPX at several time points. The increased intensity of FAOBlue fluorescence confirmed that Gcg treatment induced β-oxidation in CLPX-WT cells in a time-dependent manner (Fig. 4*A*). In parallel, CLPX protein levels were decreased to approximately 45% of those in nontreated cells at 9 h after the initiation of Gcg treatment (Fig. 4*B*). We also examined CLPX mRNA levels using quantitative real-time PCR and confirmed that Gcg treatment decreased CLPX mRNA levels after 9 h (Fig. 4*C*). These results indicate that Gcg treatment reduces CLPX protein and mRNA expression in HepG2 cells. To our knowledge, there is no report describing the regulation of CLPX protein or mRNA expression levels under physiological stimulation. To examine whether removal of Gcg from the culture medium restores CLPX levels and β-oxidation activity, we pretreated CLPX-WT cells with or without 50 nM Gcg for 9 h and then removed Gcg by replacing the culture medium with fresh culture medium (Fig. 4*D*, upper panel). As expected, the removal of Gcg from the culture medium resulted in a decrease in the fluorescence of FAOBlue in a time-dependent manner (Fig. 4*D*, lower panel and graph), and the CLPX protein levels gradually recovered (Fig. 4*E*). These results provide new insight into the regulatory conditions for CLPX protein levels and indicate that there is a strong relationship between decreases in CLPX protein levels and induction of β-oxidation in HepG2 cells during the proliferative phase.

### Mitochondrial β-oxidation–related proteins form protein complexes and are altered in CLPX-KO cells

**Figure 5:**
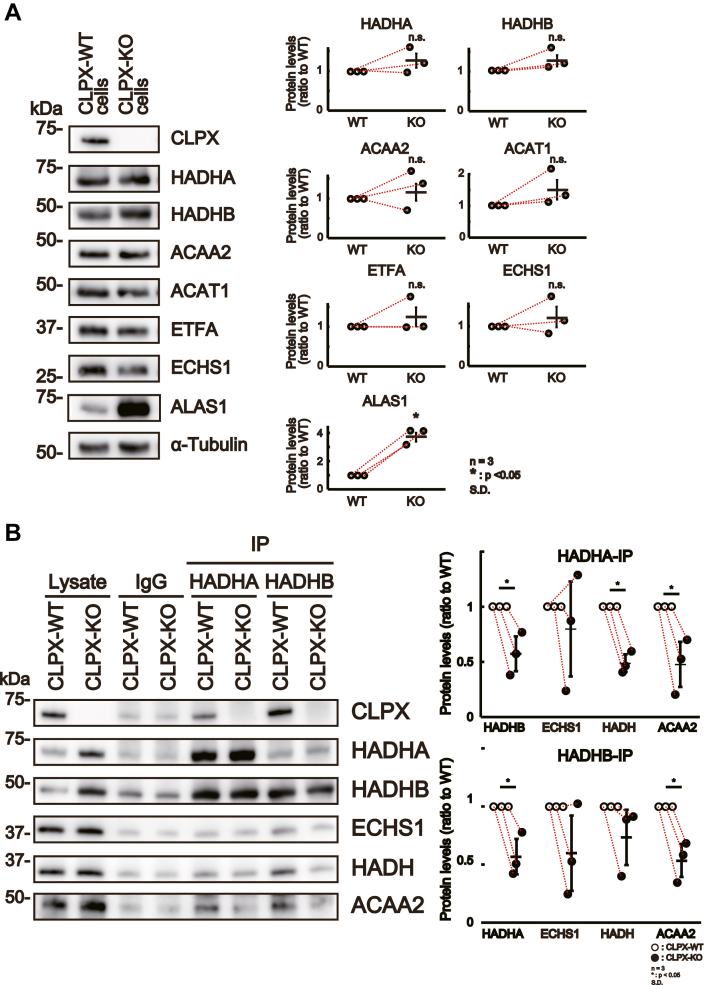
**Quantification and immunoprecipitation of β-oxidation–related proteins in CLPX-WT and CLPX-KO cells.***A*, CLPX knockout did not change the levels of proteins involved in β-oxidation. Five micrograms of protein from the total lysate of CLPX-WT or CLPX-KO cells was subjected to SDS–PAGE followed by Western blotting to determine the relative expression levels of CLPX-binding proteins involved in β-oxidation. The error bars indicate the SDs from three independent experiments. n.s. indicates “not significant” (paired *t* test). *B*, knockout of CLPX altered the relative amounts of ACAA2 among the proteins immunoprecipitated by anti-HADHA or anti-HADHB antibodies. Immunoprecipitation experiments using anti-HADHA and anti-HADHB antibodies were performed on CLPX-WT and CLPX-KO cell lysates. Rabbit IgG was used as a negative control for immunoprecipitation, and a representative result is shown in the *left panel*. The *right panels* show the quantification of the levels of the prey proteins binding to each bait protein. The error bars indicate the SDs from three independent experiments. ∗*p* < 0.05 (paired *t* test).

If CLPX regulates β-oxidation through ClpXP-mediated proteolysis, the expression levels of β-oxidation–related proteins, such as HADHA, HADHB, ACAA2, ACAT1, ETFA, and ECHS1, should be different between CLPX-WT cells and CLPX-KO cells. To clarify whether CLPX deletion affects the protein levels of β-oxidation–related proteins, we investigated the expression levels of β-oxidation–related proteins in CLPX-WT cells and CLPX-KO cells by Western blot analysis. The levels of some β-oxidation–related proteins, such as HADHA and HADHB, tended to be higher in CLPX-KO cells than in CLPX-WT cells, but the differences were not statistically significant (Fig. 5*A*). On the other hand, the protein levels of ALAS1, which is degraded by ClpXP (13), were significantly (3.7 times) higher in CLPX-KO cells than in CLPX-WT cells (Fig. 5*A*). Thus, it seems to be difficult to conclude that CLPX regulates the function of these β-oxidation–related proteins through proteolytic digestion.

Mitochondrial β-oxidation is a sequential reaction catalyzed by several specific enzymes (34). At the inner mitochondrial membrane, eight-carbon or longer fatty acids are oxidized by mitochondrial trifunctional protein (MTP), which is composed of HADHA (MTPα), HADHB (MTPβ), the β-oxidation enzymes enoyl-CoA hydratase (ECHS1 or CROT) and hydroxyacyl-CoA dehydrogenase (HADH or SCHAD), and the 3-ketoacyl-CoA thiolase (ACAA2 or MCKAT) (34). Then, shorter substrates, such as six- or four-carbon fatty acids, are sequentially oxidized by β-oxidation enzymes (ECHS1, HADH, and ACAA2) in the mitochondrial matrix (34). Among these enzymes, MTP is thought to be the hub protein complex in fatty acid oxidation (35, 36). Thus, we speculated that CLPX can modify the protein–protein interactions among these β-oxidation–related proteins, especially MTP and β-oxidation enzymes, since CLPX seems to regulate β-oxidation through its protein-binding function (Fig. 3, *B* and *C*). To test this hypothesis, we performed immunoprecipitation analysis using an anti-HADHA antibody or an anti-HADHB antibody and detected the protein levels of ECHS1, HADH, and ACAA2 in each immune complex. When HADHA was immunoprecipitated from the lysate of CLPX-KO or CLPX-WT cells, HADHB, ACAA2, and HADH proteins were significantly decreased in the immunoprecipitates of HADHA from CLPX-KO cells compared with those from CLPX-WT cells (Fig. 5*B*, right upper panel). Furthermore, HADHA and ACAA2 proteins were significantly decreased in the immunoprecipitates of HADHB from CLPX-KO cells compared with those from CLPX-WT cells (Fig. 5*B*, right lower panel). Although the finding was not statistically significant, the HADH protein level in the immunoprecipitates of HADHB from CLPX-KO cells tended to be lower than that in the immunoprecipitates from CLPX-WT cells. The ECHS1 protein levels in the immunoprecipitates of HADHA or HADHB did not significantly differ between the samples from CLPX-KO cells and those from CLPX-WT cells. These results suggest that the absence of CLPX in mitochondria results in the dissociation of HADH and ACAA2 from HADHA, dissociation of ACAA2 from HADHB, and dissociation of HADHA from HADHB in HepG2 cells. These results suggest that CLPX is involved in the formation of protein complexes containing β-oxidation–related proteins.

### Gcg treatment modify the protein complex of β-oxidation–related proteins

**Figure 6:**
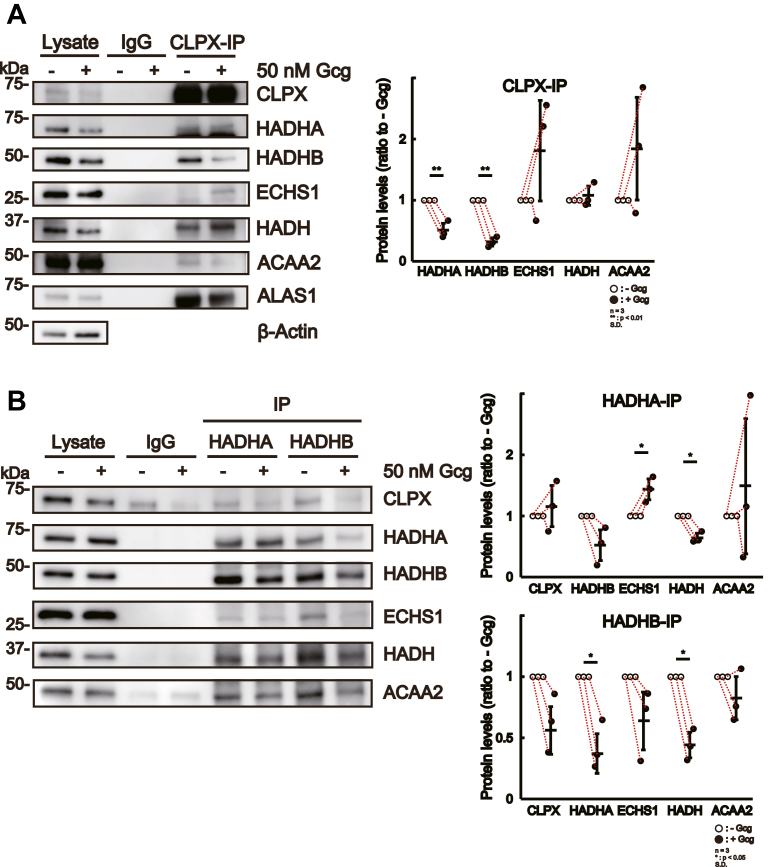
**Immunoprecipitation of CLPX or β-oxidation–related proteins in CLPX-WT or CLPX-KO cells with or without Gcg treatment.***A*, immunoprecipitation experiments using anti-CLPX antibody were performed with CLPX-WT lysates treated with or without 50 nM Gcg before immunoprecipitation. Rabbit IgG was used as a negative control for immunoprecipitation. The *left panel* shows the representative result. The *right panel* shows the results from the quantification of prey proteins binding to CLPX. The error bars indicate the SDs from three independent experiments. ∗∗*p* < 0.01 (paired *t* test). *B*, immunoprecipitation experiments using anti-HADHA and anti-HADHB antibodies were performed with CLPX-WT lysates treated with or without 50 nM Gcg before immunoprecipitation. Rabbit IgG was used as a negative control for immunoprecipitation. The *left panel* shows the representative result. The *right panels* show the results from the quantification of prey proteins binding to each bait proteins. The error bars indicate the SDs from three independent experiments. ∗*p* < 0.05 (paired *t* test). We loaded 0.5 μg of protein from each total cell lysate as an input in all panels.

As a next step, we attempted to determine whether these protein complexes containing β-oxidation–related proteins were altered in HepG2 cells after treatment with Gcg for the activation of β-oxidation. To elucidate the protein–protein interactions of endogenous CLPX protein with β-oxidation–related proteins, we performed immunoprecipitation with an anti-CLPX antibody using the lysate of CLPX-WT cells treated with or without Gcg and then subjected the immunoprecipitates to Western blot analysis. The binding of CLPX with ALAS1 was used as a positive control for the immunoprecipitation experiment, and ALAS1 was reproducibly detected in the immunoprecipitates of CLPX from Gcg-treated or untreated cells (Fig. 6*A*, left panel). Interestingly, the amounts of HADHA and HADHB proteins were significantly decreased in the immunoprecipitates of CLPX from Gcg-treated cells compared with those from untreated cells. (Fig. 6*A*, right panel). The amounts of ECHS1 and ACAA2 proteins in the immunoprecipitates of CLPX from Gcg-treated cells tended to increase after Gcg treatment, but the differences were not significant (Fig. 6*A*, right panel). These results suggest that endogenous CLPX dissociates from HADHA and HADHB after Gcg treatment.

We then examined the proteins that coimmunoprecipitated with HADHA or HADHB. In the immunoprecipitates of HADHA, the amounts of HADH proteins were significantly decreased after Gcg treatment, but those of ECHS1 proteins were significantly increased (Fig. 6*B*, right upper panel). The amounts of HADHA and HADH proteins were significantly decreased in the immunoprecipitates of HADHB from Gcg-treated cells compared with those from untreated cells (Fig. 6*B*, right upper panel). The CLPX, ECHS1, and ACAA2 protein levels in the immunoprecipitates of HADHB from Gcg-treated cells tended to be lower than those from nontreated cells, but the differences were not significant (Fig. 6*B*, right lower panel). These results suggest that Gcg treatment induces dissociation of HADH from MTPs (HADHA and HADHB) in addition to dissociation of HADHA from HADHB.

Interestingly, a decrease in the level of HADH in the immunoprecipitates of HADHA and a decrease in the level of HADHA in the immunoprecipitates of HADHB were commonly observed in CLPX-KO cells (Fig. 5*B*) and Gcg-treated CLPX-WT cells (Fig. 6*B*), both of which exhibited increased β-oxidation. Moreover, β-related enzymes (ECHS1, HADH, and ACAA2) tended to be lower in the immunoprecipitates of HADHB from CLPX-KO cells or Gcg-treated CLPX-WT cells compared with those from CLPX-WT or Gcg-untreated cells, respectively. These results suggest the possibility that CLPX is involved in the activation of β-oxidation in HepG2 cells *via* its binding to β-oxidation–related proteins.

### Knockout of the *CLPX* gene results in modification of the metabolic pathway

**Figure 7:**
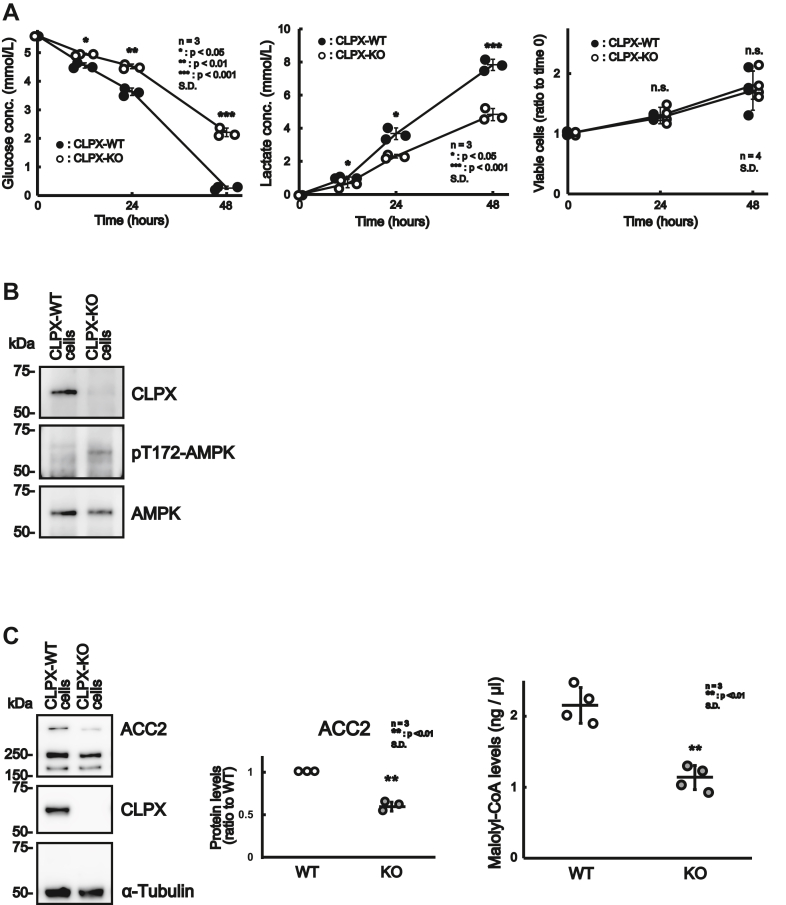
**Modification of metabolic pathways in CLPX-KO cells.***A*, CLPX-WT and CLPX-KO cells were maintained in low glucose (5.6 mM) culture medium for sampling. The glucose concentration in the culture medium of CLPX-KO cells (*open circle*) was higher than that of CLPX-WT cells (*closed circle*) at each time point (*left panel*). Consistently, the lactate concentration in the culture medium of CLPX-KO cells (*open circle*) was significantly lower than that of CLPX-WT cells (*closed circle*) at each time point (*middle panel*). The error bars indicate the SDs from three independent wells. ∗*p* < 0.05, ∗∗*p* < 0.01, and ∗∗∗*p* < 0.001 (Student’s *t* test). Cell growth of CLPX-WT cells (*right panel*, *closed circle*) and CLPX-KO cells (*right panel*, *open circle*) was examined using Cell Counting Kit-8 (CCK-8) at each time point. There were no differences in cell survival at any time point (*right panel*). The error bars indicate the SDs from four independent wells. n.s. indicates “not significant” (Student’s *t* test). *B*, to determine the phosphorylation of AMPK, the lysates of CLPX-WT and CLPX-KO cells were subjected to SDS‒PAGE and then analyzed by Western blotting using an anti-pT172 AMPK antibody. The phosphorylation of AMPK at T172 was obvious in the sample prepared from CLPX-KO cells compared to that of CLPX-WT cells. *C*, CLPX-KO cells had decreased ACC2 protein levels (*left* and *middle panel*) and malonyl-CoA levels (*right panel*) compared to CLPX-WT cells. Proteins from cell lysates of CLPX-WT cells or CLPX-KO cells were examined by Western blotting. Five micrograms of protein was subjected to SDS–PAGE followed by Western blotting. For measurement of the malonyl-CoA content, cell lysates of CLPX-WT cells or CLPX-KO cells were analyzed using a malonyl-CoA ELISA kit. AMPK, AMP-activated protein kinase.

As shown in Figure 2, *F* and *A*, glycolysis inhibitor (2-DG) suppressed ATP production in CLPX-WT cells but not in CLPX-KO cells. This result suggests that the absence of CLPX might alter the metabolic pathway in HepG2 cells. To examine the difference in glycolytic flux between CLPX-WT and CLPX-KO cells under steady-state conditions, we measured the glucose and lactate concentrations in the culture medium of CLPX-WT and CLPX-KO cells. As shown in Figure 7*A*, the consumption of glucose (left panel) and the production of lactate (middle panel) were significantly lower in CLPX-KO cells, while the number of viable cells was not different between CLPX-WT and CLPX-KO cells at each time point (right panel). Thus, these results suggest that the deletion of CLPX resulted in a decrease in glycolytic flux in HepG2 cells under steady-state conditions. However, it is unclear how the deletion of the mitochondrial protein CLPX influences glycolysis in the cytosol. Since it has been reported that AMP-activated protein kinase (AMPK) is one of the key regulators of metabolic fluxes in liver cells (37, 38) and that the activation of AMPK/mTOR signaling inhibits glycolysis (39), we determined the activation status of AMPK in these cells. As shown in Figure 7*B*, Thr172 of AMPK was phosphorylated in CLPX-KO cells compared to that in CLPX-WT cells, suggesting that AMPK is activated in CLPX-KO cells. Since the activation of AMPK also induces β-oxidation in mitochondria through the inactivation of acetyl-CoA carboxylase 2 (ACC2) or a reduction in its protein level (40), we examined the phosphorylation and expression of ACC2. Unfortunately, the antibody against phosphorylated ACC2 protein did not work well (data not shown); therefore, we determined the ACC2 protein level and the concentration of intracellular malonyl-CoA, which is the product of ACC2. As shown in Figure 7*C*, the protein expression of ACC2 and the malonyl-CoA concentration in the lysate were decreased in CLPX-KO cells. Reportedly, ACC2 is localized at the surface of mitochondria and inhibits the activity of carnitine palmitoyl transferase 1, the rate-limiting enzyme of β-oxidation, through the production of malonyl-CoA (41). Thus, these results suggest that CLPX is involved in the regulation of β-oxidation not only in mitochondria but also through the modification of the cytosolic signaling pathway, although the mechanisms for the regulation of AMPK phosphorylation remain elusive.

## Discussion

In the present study, we performed proteomic analyses using human cultured cells and successfully identified several mitochondrial proteins that bind to CLPX, a subunit of the mitochondrial protease ClpXP (Table 1). Since we used cell lysates prepared from human fibroblast cells expressing WT CLPX and CLPP for immunoprecipitation, our screening results contained the CLPX interactor as well as the substrates for the ClpXP protease. Some of the listed proteins in Table 1 have been reported as candidate ClpX-binding proteins or substrates of ClpXP in eukaryotes (20, 22, 26, 27), suggesting that our screening method worked well. Moreover, we successfully identified several proteins as novel candidates for CLPX-binding proteins, and in silico analysis enriched proteins involved in β-oxidation (Fig. 1, *B* and *C*). Interestingly, knockout of CLPX in HepG2 cells (CLPX-KO cells) resulted in an increase in β-oxidation activity without Gcg stimulation (Fig. 2*E*), and exogenous expression of CLPX attenuated the upregulation of β-oxidation activity in CLPX-KO cells (Fig. 3*B*). Knockdown of CLPX in WT HepG2 cells also resulted in an increase in β-oxidation activity (Fig. 3*E*). Consistent with the results observed in HepG2 cells, the knockdown of CLPX also activated β-oxidation activity in primary human hepatocytes (Fig. 3*G*), confirming that the increase in β-oxidation in CLPX-KO cells was not an off-target effect of genome editing. Furthermore, Gcg treatment resulted in a decrease in CLPX protein and mRNA levels in CLPX-WT cells during the proliferative phase (Fig. 4, *B* and *C*), and the removal of Gcg from the culture medium restored the CLPX levels and β-oxidation activity (Fig. 4, *D* and *E*). To our knowledge, this is the first report that describes the regulatory mechanism of endogenous CLPX protein expression in eukaryotic cells. However, the effect of Gcg on the expression level of CLPX mRNA and protein seems to be specific in HepG2 cells during the proliferative phase since we found that the expression level of CLPX did not decrease in confluently cultured HepG2 cells after Gcg treatment (Fig. S1). These results suggest that CLPX suppresses β-oxidation to maintain it at the basal level before activation by inducers, such as Gcg, in liver cells.

Immunoprecipitation experiments revealed that CLPX is involved in the protein–protein interactions of β-oxidation–related proteins (Fig. 5*B*), and Gcg treatment of HepG2 cells also altered the components of the protein complex that catalyzes β-oxidation in mitochondria and increased β-oxidation activity (Fig. 6, *A* and *B*). Importantly, immunoprecipitates of an anti-CLPX antibody contained all examined β-oxidation–related proteins (Fig. 6*A*), and immunoprecipitates of MTP protein (HADHA and HADHB) contained CLPX and all examined β-oxidation–related proteins (Fig. 6, *A* and *B*). Moreover, the deletion of CLPX expression altered the protein levels in each immunoprecipitate of HADHA or HADHB (Fig. 5*B*), and some of these alterations in the protein complex were observed in WT HepG2 cells after Gcg stimulation (Fig. 6*B*). We hypothesized that CLPX is involved in the association between MTP and β-oxidation–related proteins, and our data strongly suggest that CLPX play an important role in maintaining the levels of HADH and ACAA2 proteins in the immunoprecipitates of HADHA and for maintaining the levels of ACAA2 protein in the immunoprecipitates of HADHB (Fig. 5*B*), but whether CLPX is directly associated with these proteins remains unclear. To confirm whether CLPX is directly or indirectly associated with each β-oxidation–related protein, we need to perform several *in vitro* experiments using independently prepared recombinant proteins. We have already started to prepare several recombinant proteins, including CLPX, using bacteria for this purpose.

Importantly, both WT and E359A mutant CLPX suppressed β-oxidation activity in CLPX-KO cells, but E359A mutant-expressing CLPX-KO cells did not respond to Gcg treatment (Fig. 3*C*). Because the E359A mutant of CLPX could not release the binding protein because of the lack of ATP hydrolysis ability (16), these results suggest that the binding function of CLPX with other proteins, including β-oxidation–related proteins, is essential for the suppression of β-oxidation activity in HepG2 cells and that the release of binding proteins from CLPX might be necessary for the activation of β-oxidation as a response to Gcg treatment.

Surprisingly, the deletion of CLPX resulted in the activation of the cytosolic protein AMPK (Fig. 7*B*) and the suppression of ACC2 expression and malonyl-CoA concentration (Fig. 7*C*). Since the decrease in malonyl-CoA stimulates carnitine palmitoyl transferase 1 activity, which is the rate-limiting enzyme for the transport of fatty acids into mitochondria, CLPX is involved in the regulation of β-oxidation not only in mitochondria but also in the cytosol. The mechanisms for the activation of AMPK in CLPX-KO cells remain unknown. Reportedly, several intracellular signaling pathways are activated when mitochondrial function is impaired, such as the mitochondrial unfolded-protein response (mtUPR) (42, 43). However, we could not find a report describing the relationship between mtUPR and the deletion of CLPX, although Al-Furoukh *et al*. (19) reported that the overexpression of CLPX activated mtUPR. It is well known that increases in the intracellular AMP and ADP levels relative to the level of ATP activates AMPK (44), but it has also been reported that reactive oxygen species (ROS), such as hydrogen peroxide, activate AMPK (45, 46). Because Hinchy *et al*. (47) reported that ROS activate AMPK *via* alteration of ATP production due to mitochondrial dysfunction, it is possible that the increased production of ROS induced by activation of β-oxidation and mitochondrial dysfunction due to the absence of CLPX may induce activation of AMPK in CLPX-KO cells. The project clarifying the mechanism by which CLPX regulates the cytosolic signaling pathway, such as AMPK activation, is quite interesting, and we are now trying to identify the key molecule involved in the phosphorylation of AMPK in CLPX-KO cells.

Reportedly, clpX acts with clpP as an AAA+ protease to maintain protein homeostasis in prokaryotes (17, 48). We have also reported that CLPXP participates in negative feedback mechanisms for the regulation of heme biosynthesis to degrade ALAS1, the rate-limiting enzyme of the heme biosynthetic pathway in nonerythroid cells, in eukaryotes (13). On the other hand, Levchenko *et al*. reported that clpx is able to disassemble tetramers of Mu transposase in *Escherichia coli* without clpp (49). Since our present data indicate that CLPX regulates β-oxidation in human liver cells without degrading β-oxidation–related proteins, it is highly possible that CLPX regulates mitochondrial β-oxidation by remodeling the protein complex that consists of β-oxidation–related proteins. Such a function of clpx has been proposed by Burton and Baker (50).

Recently, nonalcoholic fatty liver disease (NAFLD) and its advanced pathological phenotype, nonalcoholic steatohepatitis (NASH), have been recognized as common and important liver diseases that could be associated with hepatocellular carcinoma (51, 52). Considering lipid accumulation in the liver as a causative condition of NAFLD, the activation of β-oxidation in the liver might be useful for the prevention of NAFLD and NASH. Although the relationship between β-oxidation and these liver diseases is complicated, some papers report reduced β-oxidation in patients with NAFLD or NASH (53, 54). Thus, the chemical modifier of CLPX function could become a promising candidate for therapeutic targets of NAFLD and NASH.

It is also interesting that ClpP KO mice are viable and are protected from diet-induced obesity and insulin resistance (28, 29), whereas knockout of the *ClpX* gene in mice results in an embryonic lethal phenotype (2), suggesting that ClpX can play an essential role at a specific stage during the development of embryos without ClpP. Based on our data, the absence of ClpX did not affect the expression levels of β-oxidation–related enzymes (Fig. 5*A*), and the suppression of CLPP using siRNA did not result in the activation of β-oxidation but rather suppressed it in liver cells (Fig. 3, *D* and *E*). Thus, we believe that ClpX regulates β-oxidation by remodeling the protein complex of β-oxidation enzymes in liver cells; however, it is important to clarify the role of ClpX in β-oxidation *in vivo* by analyzing liver-specific ClpX KO mice.

In conclusion, our present study revealed several CLPX-binding proteins by LC–MS/MS. Importantly, we found that CLPX associates with several enzymes catalyzing β-oxidation in mitochondria and that CLPX regulates β-oxidation activity in human liver cells by remodeling a protein complex that catalyzes β-oxidation at the mitochondrial inner membrane and matrix. Moreover, the deletion of CLPX resulted in the activation of AMPK, which is a well-known switch in the metabolic pathway. Future studies will further clarify how Gcg regulates the expression and function of CLPX in liver cells and determine the role of CLPX in eukaryotic fatty acid β-oxidation.

## Experimental procedures

### Reagents

Unless otherwise noted, all chemicals were purchased from Sigma–Aldrich, FUJIFILM-Wako Pure Chemical Corporation, and Nacalai Tesque. Anti-DDDDK-agarose and DDDDK peptides for the purification of FLAG-tagged proteins were purchased from Medical and Biological Laboratories Co, Ltd (MBL). The following antibodies were used for Western bot analysis: anti-ACAA2 (1:2,000, Proteintech, #11111-1-AP), anti-ACAT1 (1:2,000, Proteintech, #16215-1-A), anti-ALAS1 (1:2,000, Abcam, ab154860), anti-αTubulin pAb-HRP-DirecT (1:5,000, MBL, #PM054-7), anti-CLPP (1:3,000, Abcam, #ab124822), anti-CLPX (1:2,000, Abcam, #ab168338), anti-ECHS1 (1:2,000, Proteintech, #11305-1-AP), anti-ETFA (1:1,000, GeneTex, #GTX105155), anti-FLAG M2 Monoclonal Antibody-Peroxidase Conjugate (1:5,000, Sigma–Aldrich, #A8592), anti-HADH (1: 2,000, GeneTex, #GTX105167), anti-HADHA (1:2,000, Abcam, #ab203114), anti-HADHB (1:2,000, Bethyl Laboratories, Inc, #A305-021A), anti-LONP1 (1:2,000, Sigma–Aldrich, #HPA002192), anti-pT197-PKA (1:2,000, Cell Signaling Technology, #5661), anti-total-PKA (1:2,000, Cell Signaling Technology, #5842), anti-pS473-Akt (1:2,000, Cell Signaling Technology, #9271), anti-total-Akt (1:2,000, Cell Signaling Technology, #4691), anti-total-ACC2 (1:1,000, Cell Signaling Technology, #8578), anti-pT172-AMPK (1:2000, GeneTex, #GTX130429), anti-total-AMPK (1:2000, GeneTex, #GTX132674), HRP-conjugated anti-rabbit IgG (1:2000-3,000, Cell Signaling Technology, #7074), and rabbit TrueBlot: HRP-conjugated anti-rabbit IgG (1:2,000, Rockland Immunochemicals, Inc, #1808816-31).

### Cell culture

Flp-In T-REx 293 (FT293) cells and HepG2 cells were maintained in high-glucose Dulbecco’s modified Eagle’s medium (FUJIFILM-Wako Pure Chemical Corporation) supplemented with 10% fatal bovine serum (FBS), 50 units/ml penicillin, and 50 μg/ml streptomycin. The expression of FLAG-tagged proteins in OTC-Lucf^ind^/FT293 and CLPXf^ind^/FT293^△CLPX^ cells was induced by the addition of 1 μg/ml doxycycline to the culture medium, and the cells were incubated for 48 h. For transient expression of CLPXf-WT or CLPXf-E359A in CLPX-KO cells, the pcDNA5 plasmid vector containing an expression cassette for CLPXf-WT or CLPXf-E359A was introduced into CLPX-KO cells using Lipofectamine 3000 Reagent (Thermo Fisher Scientific) according to the manufacturer’s instructions. The transfected cells were maintained in the culture medium for 72 h before treatment or sample preparation. Human hepatocytes were purchased from ScienCell Research Laboratories (#5200) and maintained in Hepatocyte Medium (ScienCell Research Laboratories, #5201) according to the manufacturer’s instructions.

### Immunoprecipitation and Western blot analysis

For immunoprecipitation analysis, 5 x 10^6^ HepG2 cells were seeded in 150 mm culture dishes. Cells were lysed in lysis buffer (20 mM Hepes, pH 7.5, 150 mM NaCl, 1% Triton X-100, 10% glycerol, cOmplete EDTA-free protease inhibitor cocktail, 1 mM EDTA, pH 8.0, 1 mM NaF, and 0.4 mM Na_3_VO_4_) by pipetting. For Western blot analysis with anti-phosphorylation antibodies, RIPA buffer (50 mM Tris–HCl, pH 7.5, 150 mM NaCl, 1% NP40, 0.5% sodium deoxycholate, 0.1% SDS, 1 mM EDTA, 1 mM NaF, and 0.4 mM Na_3_VO_4_) or lysis buffer (20 mM Hepes, pH 7.5, 100 mM NaCl, 1 mM MgCl_2_, 0.5% NP40, and cOmplete EDTA-free protease inhibitor cocktail) was used to prepare protein samples (55). The lysed cells were centrifuged at 18,000*g* for 15 min at 4 °C. Then, the supernatant was collected and used as the cell lysate. The protein concentration of the cell lysate was determined using Pierce 660 nm protein assay reagent (Thermo Fisher Scientific) using bovine serum albumin (BSA) as a standard.

FLAG-tagged proteins were purified using anti-DDDDK-agarose beads and eluted using DDDDK peptides. Generally, 2 mg of cell lysate was subjected to immunoprecipitation experiments. Cell lysate containing the FLAG-tagged protein was incubated with the beads for 3 h at 4 °C with gentle rotation. The beads were washed with wash buffer (20 mM Hepes, pH 7.5, 150 mM NaCl, 0.1% Triton X-100, and 10% glycerol) three times and then incubated with 0.1 mg/ml DDDDK peptides for 30 min at 4 °C with gentle rotation. Each sample was centrifuged at 18,000*g* for 5 min at 4 °C. The supernatant was collected as an eluent containing the immunoprecipitated proteins. For immunoprecipitation of specific proteins, cell lysate was incubated with 1 μg of each specific antibody for 1 h on ice, and then 5 μl (10% slurry) of Dynabeads Protein G for immunoprecipitation (Thermo Fisher Scientific, #1003D) was added to the sample. The sample was incubated overnight at 4 °C with gentle rotation. The beads were washed with wash buffer (20 mM Hepes, pH 7.5, 150 mM NaCl, 0.1% Triton X-100, and 10% glycerol) three times and then incubated with 7.5 μl of 2× SDS–PAGE sample buffer at 95 °C for 5 min to elute the binding proteins from the magnetic beads.

For Western blot analysis, the final protein concentration was adjusted to 1 μg/μl using lysis buffer, and samples were incubated with 6× concentrated SDS–PAGE sample buffer (Nacalai Tesque, #09499-14) at 95 °C for 5 min. Protein samples (0.5–10 μg) were loaded onto a 10 or 12% TGX acrylamide gel (Bio-Rad Laboratories Inc) and electrophoresed. Then, the proteins were transferred to polyvinylidene difluoride membranes with a Trans-Blot Turbo system (Bio-Rad) according to the manufacturer’s instructions. After blotting, the polyvinylidene difluoride membranes were blocked with Tris-buffered saline with 0.05% Tween 20 (TBS-T) containing 5% skimmed milk (w/v) for 1 h at room temperature. When antibodies that recognize phosphorylated amino acids were used, the membranes were blocked with TBS-T containing 3% BSA (w/v) for 1 h at room temperature or overnight at 4 °C. The membranes were incubated with specific primary antibodies diluted in TBS-T or Can Get Signal Immunoreaction Enhancer Solution (TOYOBO) for 1 h at room temperature or overnight at 4 °C and washed with TBS-T three times for 7 min. Then, the membranes were incubated with HRP-conjugated secondary antibodies for 1 h at room temperature and washed with TBS-T three times for 7 min. The signals were detected using Clarity ECL Western substrate (Bio-Rad), and images were obtained using an ImageQuant LAS500 image analyzer (GE Healthcare). For the quantification of protein expression, the signal intensities of proteins of interest were measured using ImageJ software (https://imagej.nih.gov/ij/). The signal intensities of proteins of interest were then normalized using those of α-tubulin as an internal control.

For the quantification of coimmunoprecipitates of HADHA or HADHB, we performed immunoprecipitation experiments using three pairs of CLPX-WT and CLPX-KO cells or three pairs of Gcg-treated and untreated CLPX-WT cells. We loaded all the samples in the same polyacrylamide gel and performed Western blotting. We then measured the signal intensities of prey proteins of interest and normalized these using the signal intensities of the bait protein. The normalized data of the prey proteins in the immunoprecipitates of each bait protein from CLPX-KO or Gcg-treated CLPX-WT cells were compared with those in the immunoprecipitates from paired CLPX-WT or untreated control cells, respectively. The results from CLPX-KO cells or Gcg-treated cells are expressed as ratios to those of control cells. The blots used for the quantification of coimmunoprecipitated proteins are shown as Figs. S2–S4.

### Quantitative real-time PCR

Total RNA was extracted from HepG2 cells using Isogen II reagent (Nippon Gene). The concentration of each purified RNA sample was quantified using a UV-1800 spectrophotometer (Shimadzu), and 1 μg of RNA was used for the reverse transcriptase reaction using a PrimeScript RT Reagent Kit with DNA Eraser (Takara Bio). Quantitative real-time PCR was performed as described previously (56).

### LC–MS/MS analysis and bioinformatic analysis

FLAG-tagged proteins were immunoprecipitated as described above. The protein concentration of the immunoprecipitate eluent was determined with a NanoDrop 2000 (Thermo Fisher Scientific). Two micrograms of each immunoprecipitated protein was digested with Sequencing Grade Modified Trypsin (Promega, #V5111) as described previously (57) with some modifications. Each sample (15 μl/45 μl) was injected into a Zaplous U3000-PAL System (AMR, Inc) connected to an L-column ODS (0.1 × 150 mm, Chemicals Evaluation and Research Institute). The peptides were eluted with a 180-min gradient of 5 to 45% solvent B (0.1% TFA in 90% acetonitrile with 9.9% H_2_O, v/v) in solvent A (0.1% TFA and 2% acetonitrile in H_2_O, v/v) at a flow rate of 300 nl/min. The peptides were then ionized and analyzed using an LTQ Orbitrap XL ETD (Thermo Fisher Scientific). The MS/MS spectra data were obtained from three independent experiments. The MS/MS data were analyzed as described previously with some modifications (13). The parameters used to identify the protein sequences in MASCOT with the UniProt protein database included carbamidomethylation of cysteine as a fixed modification and oxidation of methionine as a variable modification. The other parameters included one allowed missed cleavage, a mass error of 10 ppm for precursor ions, and a mass error of 0.6 Da for fragment ions. Identified proteins were selected according to a threshold of at least two unique peptides in at least two of the three experiments. For the analysis conducted with DAVID Bioinformatics Resources v.6.8, the UniProt form of the dataset for each FLAG-tagged protein was submitted and then analyzed with the UniProt ID as the identifier and Gene List as the list type. Mitochondrial proteins were extracted from the Functional Annotation Chart of UP_KEYWORDS, Mitochondrion. The classified mitochondrial protein list was further analyzed as described above. Specific intracellular functions were predicted according to annotation clusters obtained from functional annotation clustering. Categories with FDR values < 0.05 were selected as predicted functions for each protein.

### FAOBlue analysis

FAOBlue reagent was purchased from Funakoshi Co, Ltd, (FDV-0033) (30). The day before the FAOBlue reaction, the culture medium of HepG2 cells was replaced with high-glucose Dulbecco’s modified Eagle’s medium without FBS, and the cells were incubated for 12 h. The next day, the culture medium was replaced with fresh culture medium with/without 50 nM Gcg or 50 nM Ins for the indicated times. After the replacement of the culture medium with HBS buffer (20 mM Hepes, pH 7.5, 107 mM NaCl, 6 mM KCl, 1.2 mM MgSO_4_, 2 mM CaCl_2_, and 11.5 mM glucose), a final concentration of 10 μM FAOBlue reagent was added, and the cells were further incubated for 30 min at 37 °C and 5% CO_2_ before the detection of fluorescence. A FAOBlue fluorescent image (Ex. 403 nm/Em. 460 nm) and differential interference contrast (DIC) images were obtained using an A1R HD25 confocal laser scanning microscope (Nikon Co.) or an IN CELL Analyzer 2000 (Cytiva). The fluorescence of FAOBlue was quantified with IN CELL Developer (Cytiva). Since the DIC image itself was not suitable for quantification, it was translated into a pseudofluorescence image, and the DIC total area (DIC-area) was determined. Then, β-oxidation activity was determined based on the intensity of the FAOBlue fluorescence image (FAO-int). The mean intensity of β-oxidation was determined automatically according to the FAO-int per DIC area by using the IN CELL Developer algorithm.

### Knockdown or forced expression of genes

For the knockdown experiments, Silencer Select siRNAs (Thermo Fisher Scientific) were introduced into the cells. CLPX-WT HepG2 cells were seeded into 24-well plates, and the cells were transfected with 10 nM siRNA targeting *CLPP* (5′-CCCGUAUCAUGAUCCACCATT-3′), *CLPX* (5′-GGCUGGAUAUGUAGGCGAATT-3′), and *LONP1* (5′-AGCUAAAGAUCAUCAAGAATT-3′) using Lipofectamine RNAiMAX Transfection Reagent (Thermo Fisher Scientific) according to the manufacturer’s instructions. Seventy-two hours after transfection, the culture medium was changed to medium without FBS. Twelve hours later, β-oxidation was detected as described above, and the cells were harvested to prepare cell lysates for Western blot analysis.

Human primary hepatocytes were seeded into 96-well plates. siRNAs were transfected as described above for 96 h (first transfection). Then, siRNAs were transfected again and maintained for 80 hours (second transfection). A total of 180 h after the first transfection, the culture medium was changed to medium without FBS. Twelve hours later (total of 192 h after the first transfection), β-oxidation was detected as described above, and the cells were harvested to prepare cell lysates for Western blot analysis.

For the transient expression of exogenous CLPX, we used a pcDNA5/FRT/TO vector that contains complementary DNA (cDNA) encoding C-terminally FLAG-tagged WT or mutant-type (E359A) CLPX. The empty vector was used as a negative control. One to 10 μg of plasmid vector was transfected using Lipofectamine 3000 Reagent (Thermo Fisher Scientific) according to the manufacturer’s instructions. Sixty hours after transfection, the culture medium was changed to medium without FBS. Seventy-two hours after transfection, β-oxidation was detected as described above, and cells were harvested to prepare cell lysates for Western blot analysis.

### Establishment of CLPX-KO cells and OTC-LucF cells

The establishment of FT293^△CLPX^ cells has been previously described (13). For the establishment of CLPX-KO cells, the PX459-CLPX plasmid (13) was introduced into HepG2 cells, and the cells were seeded after limited dilution. Clones in which CLPX expression was decreased or absent were selected by Western blot analysis. For the establishment of OTC-LucF cells, human *OTC* cDNA (NM_000531.5) encoding an OTC precursor protein was amplified by PCR with the following primers: 5′-GAATTCAAGATGCTGTTTAATCTGAGG-3′ and 5′- GTCGACAAATTTAGGCTTCTGGAGCTG-3′. The amplified products were cloned into the pMD20-T vector (Takara Bio). The resultant plasmid was used as a template for PCR using primers 5′-GAATTCAAGATGCTGTTTAATCTGAGG-3′ and 5′-CCCGGGTTGTAGTGGTTGTCCACACCGA-3′ to amplify the cDNA sequence corresponding to the mitochondrial transfer signal of the OTC (1–32 amino acids) (58). Then, the PCR product was digested with EcoRI and XmaI and cloned into the pEGFPN2 vector (BD Bioscience) with a luciferase-FLAG gene, which was derived from the plasmid described previously (59). After the nucleotide sequence of the OTC was checked, the plasmid was digested with EcoRI and then ligated with EcoRI-NotI adapter oligonucleotide. The OTC-Luciferase-FLAG fragment, which was obtained by digestion of the product of the ligation reaction with NotI, was cloned into the pcDNA5/FRT/TO vector and referred to as the OTC-LucF plasmid.

### Genotyping of CLPX-KO cells

For the genotyping of CLPX-KO cells, the locus targeted by Cas9 in CLPX-KO cells was amplified by PCR (KOD -Multi & Epi-, TOYOBO), and the PCR products were ligated with the pGEM-T Easy vector (Promega). The ligation mixture was used to transform *E. coli*, and independent colonies were picked for colony PCR. The colony PCR products were used as a template for Sanger sequencing, and the sequence was determined by an ABI3500 Genetic Analyzer (Applied Biosystems). The sequences of the primer set for PCR were as follows: 5′-GTGAACTCTCCACGGGGTCTA-3′ and 5′-ACAGGAGCATTTTGTGCCTTTC-3′. For colony PCR and Sanger sequencing, T7 (5′-TAATACGACTCACTATAGGG-3′) and SP6 (5′-ATTTAGGTGACACTATAGAA-3′) primers were used. PCR products were purified using the Monarch DNA Gel Extraction Kit (New England Biolabs).

### Quantification of oxygen consumption and ATP levels

Cells were cultured in the presence or absence of 25 mM 2-DG (2-DG, Sigma‒Aldrich, D8375-10 MG) for 12 h. Then, oxygen consumption was measured by an Oxygen Consumption Rate Assay Kit (Cayman, #600800) using a Tecan Spark microplate reader (Tecan Group Ltd) according to the manufacturers’ instructions. To determine the background, cells were treated with 1 mM antimycin A. ATP levels were determined by using Cell Titer-Glo 2.0 Cell Viability Assay (Promega, #G9241) according to the manufacturer’s instructions.

### Quantification of malonyl-CoA by ELISA

Cells were lysed in PBS by pipetting, and cells were frozen and thawed in liquid nitrogen three times. For the determination of protein concentration, 2% CHAPS was added to the cell lysate, and the protein concentration was determined using Pierce 660 nm protein assay reagent (Thermo Fisher Scientific) using BSA as a standard. Fifty micrograms of protein was used for the Human Malonyl Coenzyme A ELISA Kit (MyBioSource, #MBS705079). ELISA experiments were performed according to the manufacturer’s instructions.

### Quantification of extracellular glucose, lactate, and cell survival

HepG2 cells (5 × 10^4^ cells/well) were cultured in 48-well plates. Extracellular glucose and lactate were quantified using a Glucose Assay Kit-WST (Dojindo Laboratories, #G264) and a Lactate Assay Kit-WST (Dojindo Laboratories, #L256), respectively. Experiments were performed according to the manufacturer’s instructions. Briefly, 4 out of 200 μl of cultured medium were diluted in 196 μl of ultrapure water. Fifty or twenty microliters of diluted medium was used for the Glucose Assay Kit-WST or Lactate Assay Kit-WST, respectively. Samples were collected serially from the same wells at 12, 24, and 48 h after medium change. Cell survival was quantified by using Cell Counting Kit-8 (Dojindo Laboratories, #CK04) according to the manufacturer’s instructions.

### Statistical analysis

All results are presented as the means ± SDs from at least three independent experiments. All two-group comparisons were performed with unpaired two-tailed Student’s t tests, and *p* < 0.05 was considered to indicate statistical significance. All comparisons of more than two groups were performed with one-way ANOVA with Tukey’s post hoc test, and *p* < 0.05 was considered to indicate statistical significance.

## Data availability

All data are contained in the manuscript.

## Supporting information

This article contains supporting information.

## Conflict of interest

The authors declare that they have no conflicts of interest with the contents of this article.
