# Supplementary material for: CLPX regulates mitochondrial fatty acid β-oxidation in liver cells
Source: J Biol Chem. 2023 Sep 3;299(10):105210. doi: 10.1016/j.jbc.2023.105210 (PMC10556790; doi:10.1016/j.jbc.2023.105210)
Supplement: Supporting information [file mmc1.docx]

**Figure legends**

**Supplementary Figure 1. Immunoprecipitation of CLPX or β-oxidation related proteins in CLPX-WT with or without Gcg treatment under confluent condition.**

**a** Immunoprecipitation experiments using anti-HADHA and anti-HADHB antibodies were performed on CLPX-WT lysates treated with or without 50 nM Gcg before immunoprecipitation. Rabbit IgG was used as a negative control for immunoprecipitation. **b** Immunoprecipitation experiments using anti-CLPX antibody were performed on CLPX-WT lysates treated with or without 50 nM Gcg before immunoprecipitation. Rabbit IgG was used as a negative control for immunoprecipitation. **c** Immunoprecipitation experiments using anti-ACAA2 antibody were performed on CLPX-WT lysates treated with or without 50 nM Gcg before immunoprecipitation. Rabbit IgG was used as a negative control for immunoprecipitation.

**Supplementary Figure 2**

**a** Western blot data which was used for the quantification of immunoprecipitation experiments using an anti-HADHA antibody between CLPX-WT and CLPX-KO cell lysates. **b** Western blot data which was used for the quantification of immunoprecipitation experiments using an anti-HADHB antibody between CLPX-WT and CLPX-KO cell lysates.

**Supplementary Figure 3**

Western blot data which was used for the quantification of immunoprecipitation experiments using anti-CLPX antibody on CLPX-WT lysates treated with or without 50 nM Gcg before immunoprecipitation.

**Supplementary Figure 4**

**a** Western blot data which was used for the quantification of immunoprecipitation experiments using anti-HADHA antibody on CLPX-WT lysates treated with or without 50 nM Gcg before immunoprecipitation. **b** Western blot data which was used for the quantification of immunoprecipitation experiments using anti-HADHB antibody on CLPX-WT lysates treated with or without 50 nM Gcg before immunoprecipitation. It should be noted that order in lysate lanes are opposite from them of immunoprecipitation experiments using anti-HADHA antibody.
