# Supplementary figures and images for: CLPX regulates mitochondrial fatty acid β-oxidation in liver cells
Source: J Biol Chem. 2023 Sep 3;299(10):105210. doi: 10.1016/j.jbc.2023.105210 (PMC10556790; doi:10.1016/j.jbc.2023.105210)

# Supplementary Figure 1

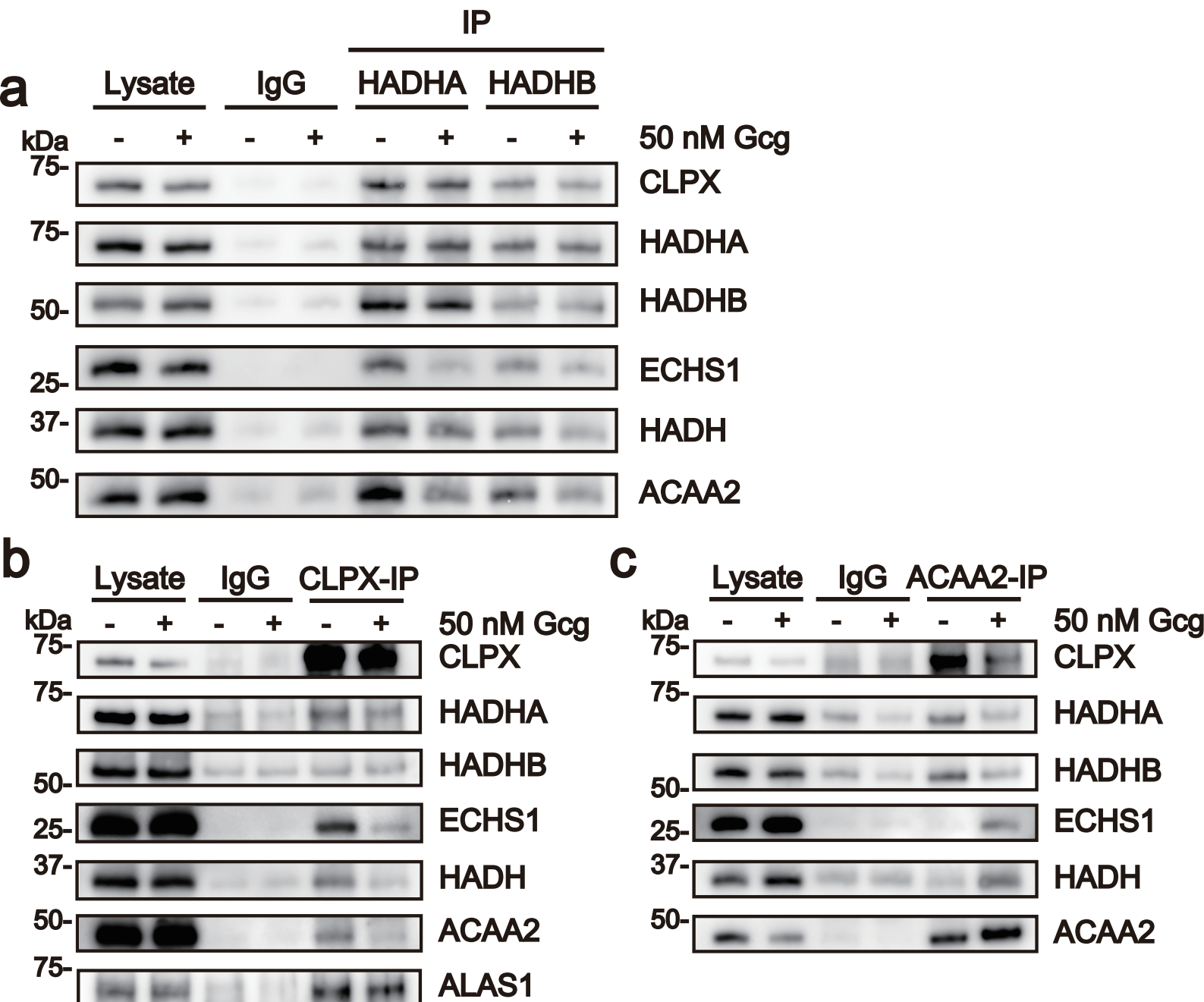

Supplement: Figure S1 [file mmc2.pdf]

# Supplementary Figure 2

**a**

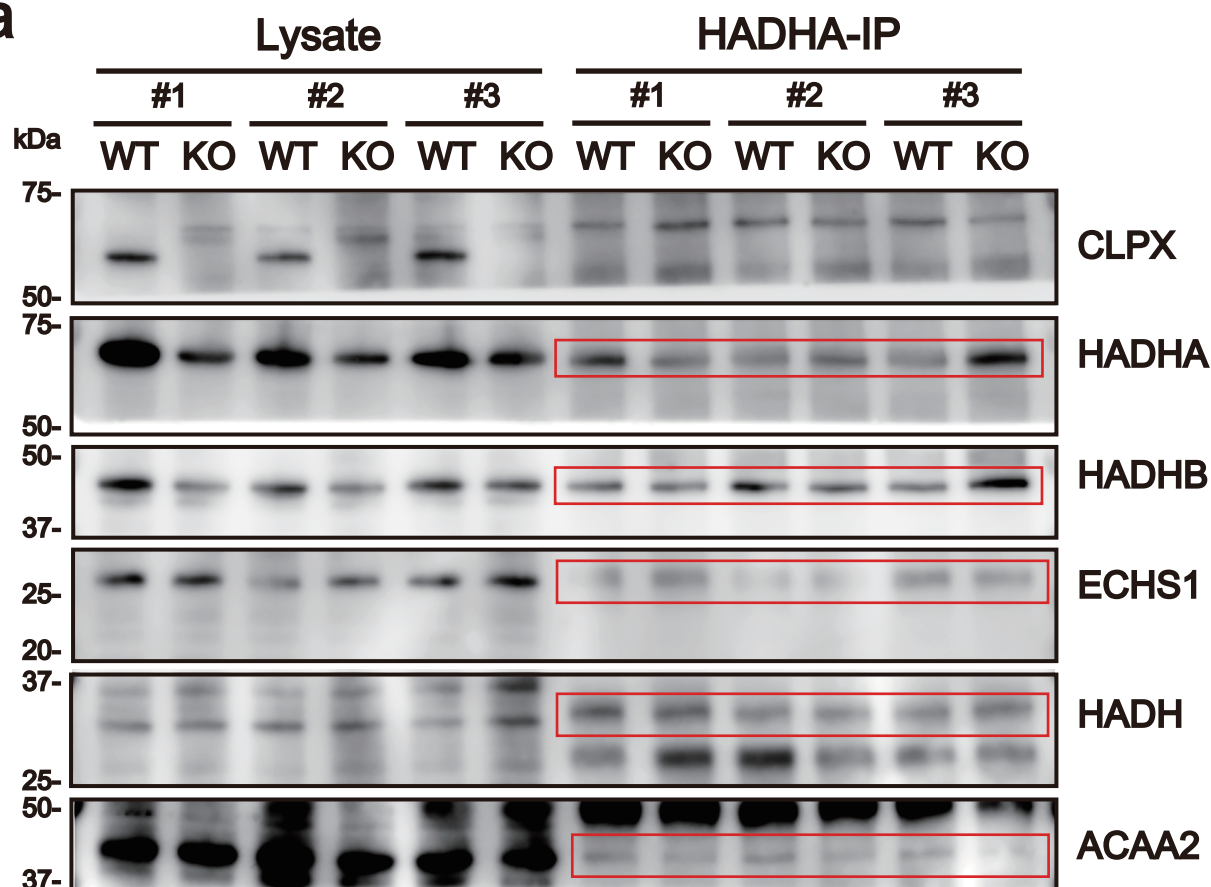

**b**

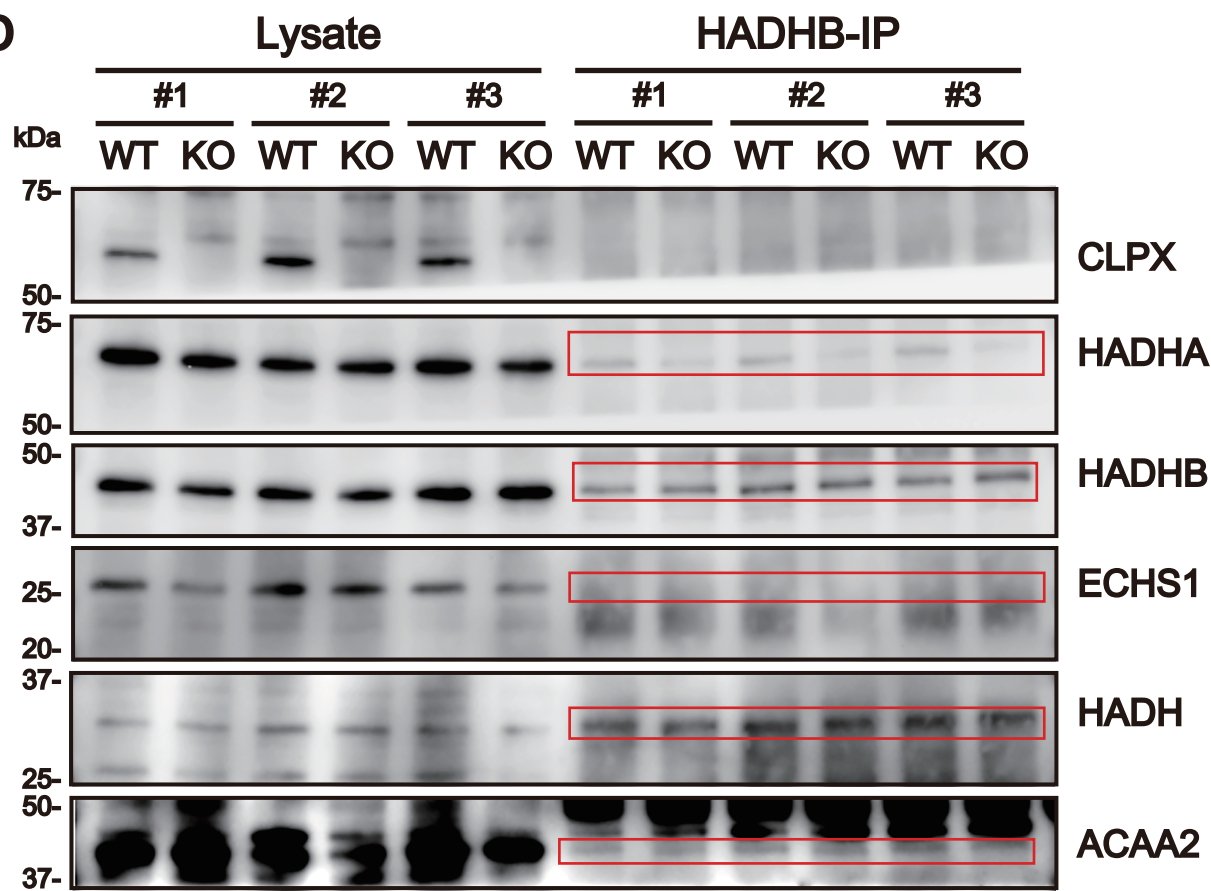

Supplement: Figure S2 [file mmc3.pdf]

# Supplementary Figure 3

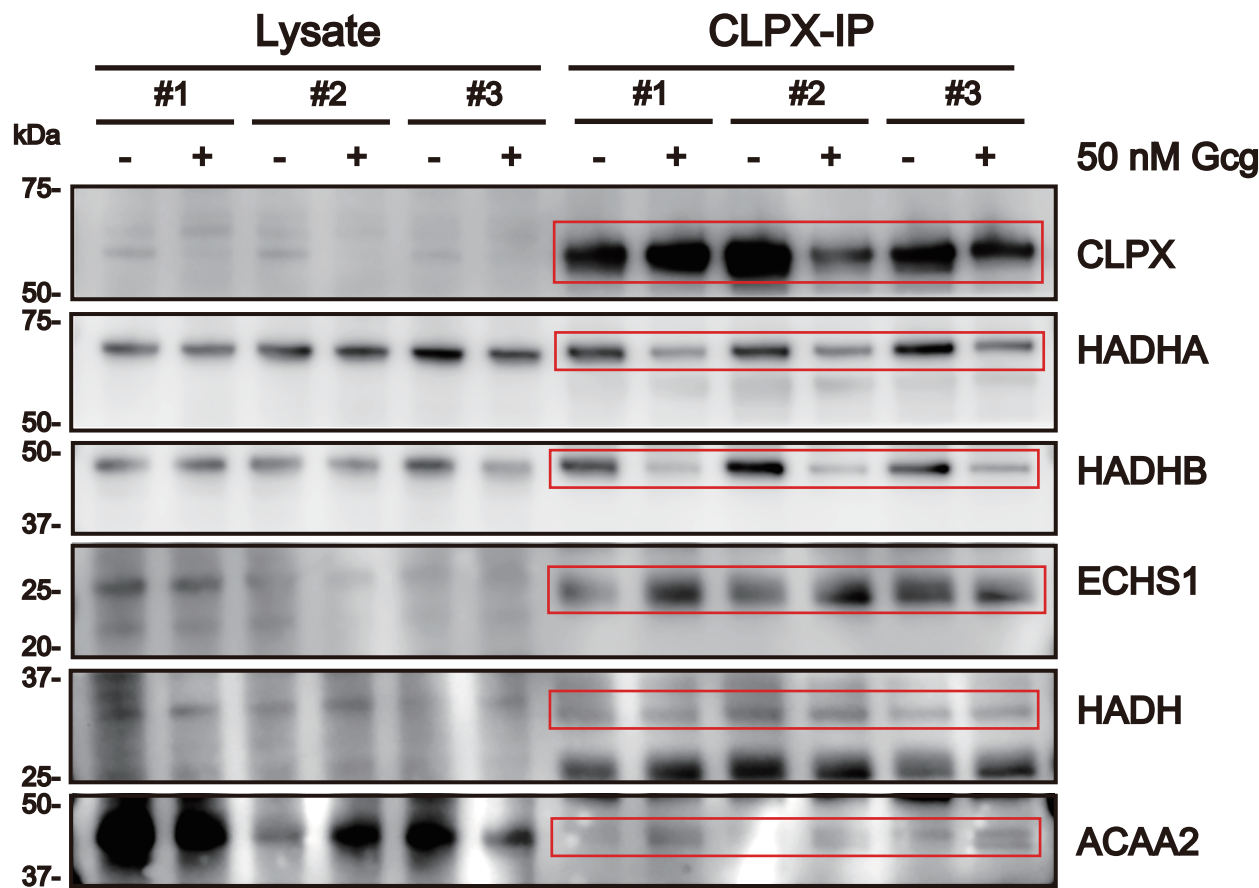

Supplement: Figure S3 [file mmc4.pdf]

# Supplementary Figure 4

**a**

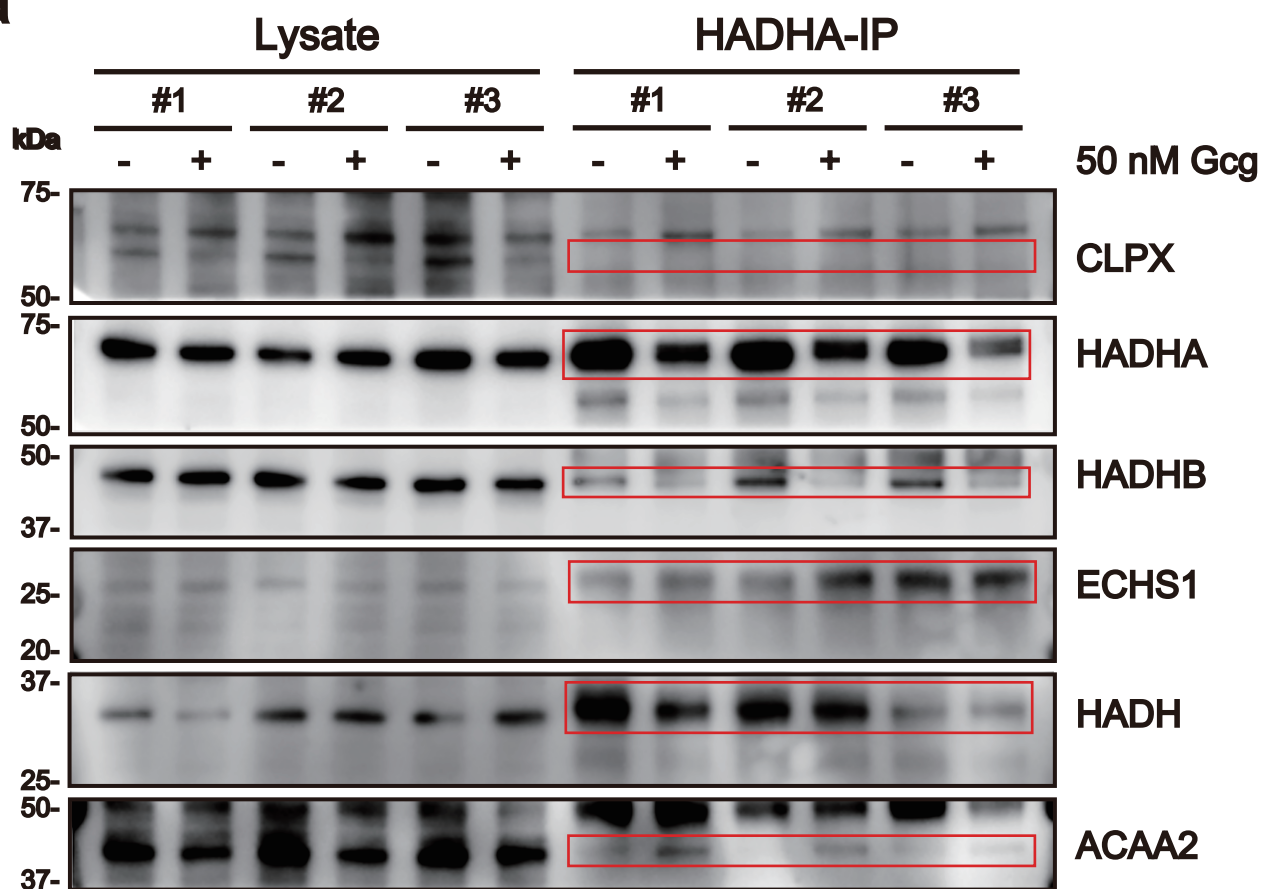

**b**

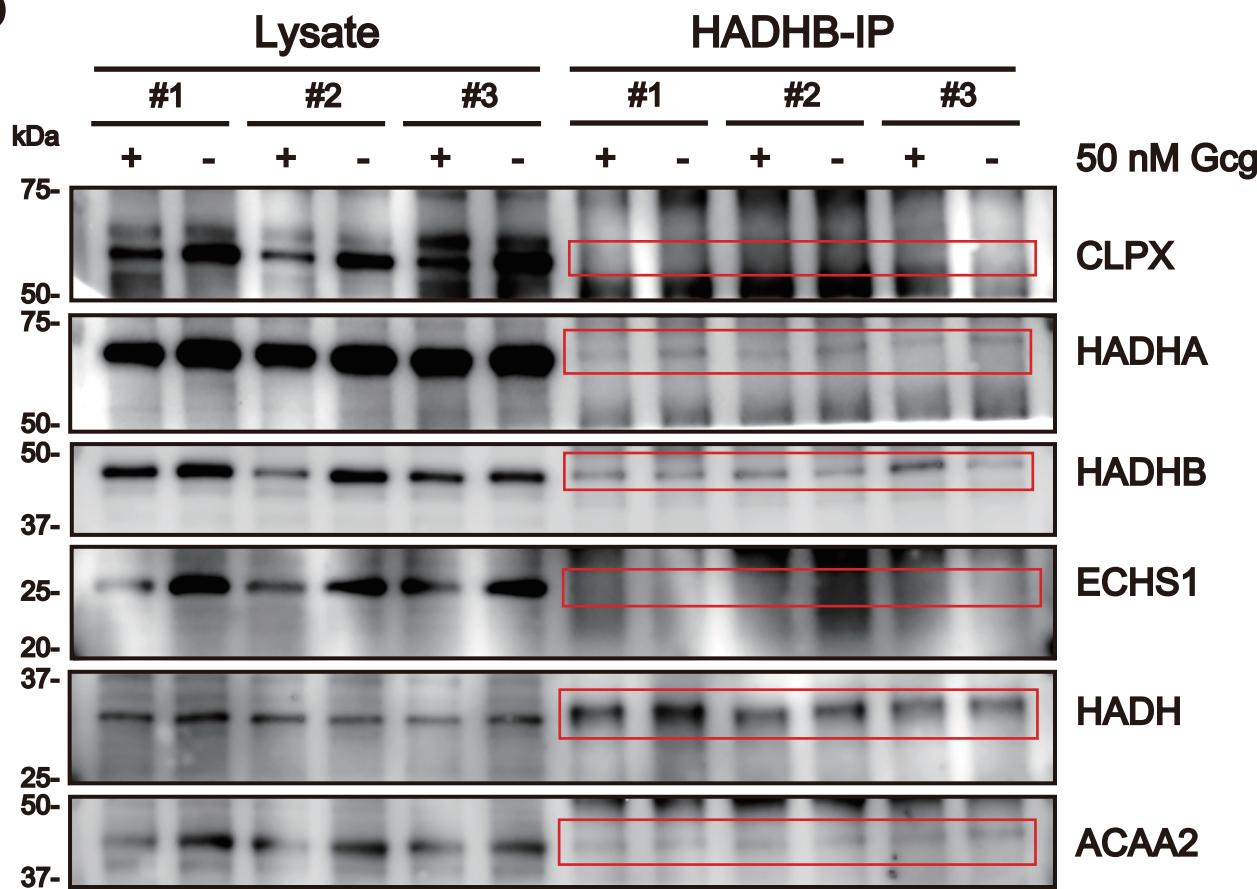

Supplement: Figure S4 [file mmc5.pdf]
